# Traditional herbs: mechanisms to combat cellular senescence

**DOI:** 10.18632/aging.205269

**Published:** 2023-12-05

**Authors:** Lei Wang, Jiahui Wang, Zhihui Yang, Yue Wang, Tiejian Zhao, Weisheng Luo, Tianjian Liang, Zheng Yang

**Affiliations:** 1 Graduate School, Guangxi University of Chinese Medicine, Nanning, Guangxi 530222, China; 2 Department of Medicine, Faculty of Chinese Medicine Science Guangxi University of Chinese Medicine, Nanning, Guangxi 530222, China; 3 Department of Physiology, College of Basic Medicine, Guangxi University of Chinese Medicine, Nanning, Guangxi 530222, China; 4 Ruikang Hospital Affiliated to Guangxi University of Chinese Medicine, Nanning, Guangxi 530000, China

**Keywords:** Chinese herbal medicine, cellular senescence, inflammatory response, intestinal flora

## Abstract

Cellular senescence plays a very important role in the ageing of organisms and age-related diseases that increase with age, a process that involves physiological, structural, biochemical and molecular changes in cells. In recent years, it has been found that the active ingredients of herbs and their natural products can prevent and control cellular senescence by affecting telomerase activity, oxidative stress response, autophagy, mitochondrial disorders, DNA damage, inflammatory response, metabolism, intestinal flora, and other factors. In this paper, we review the research information on the prevention and control of cellular senescence in Chinese herbal medicine through computer searches of PubMed, Web of Science, Science Direct and CNKI databases.

## INTRODUCTION

Cellular senescence is when a cell enters permanent cell cycle arrest and is unable to re-enter the cell cycle in response to various known stimuli. Cellular senescence, a state of permanent cell cycle termination, is one of the main hallmarks of ageing, and the main feature of senescent organs is the accumulation of senescent cells [1]. Stem cells play a crucial role in maintaining the regenerative capacity of normal tissues in the body. However, they are susceptible to various factors that can deplete their numbers, ultimately impacting the overall aging process of the body [2]. Cellular senescence is a cellular state that is induced by stress signals and exists in a specific physiological process, with four primary characteristics that are characteristic of cell cycle arrest, senescence-related secretory phenotypes, macromolecular damage, and metabolic problems [3]. Cellular senescence involves a variety of molecular mechanisms and can be caused by cellular stalling due to telomere length shortening, oxidative stress, autophagy dysregulation, mitochondrial disorders, DNA damage, inflamma-tory responses, metabolism, and dysregulation of the gut flora [4].

**Figure 1:**
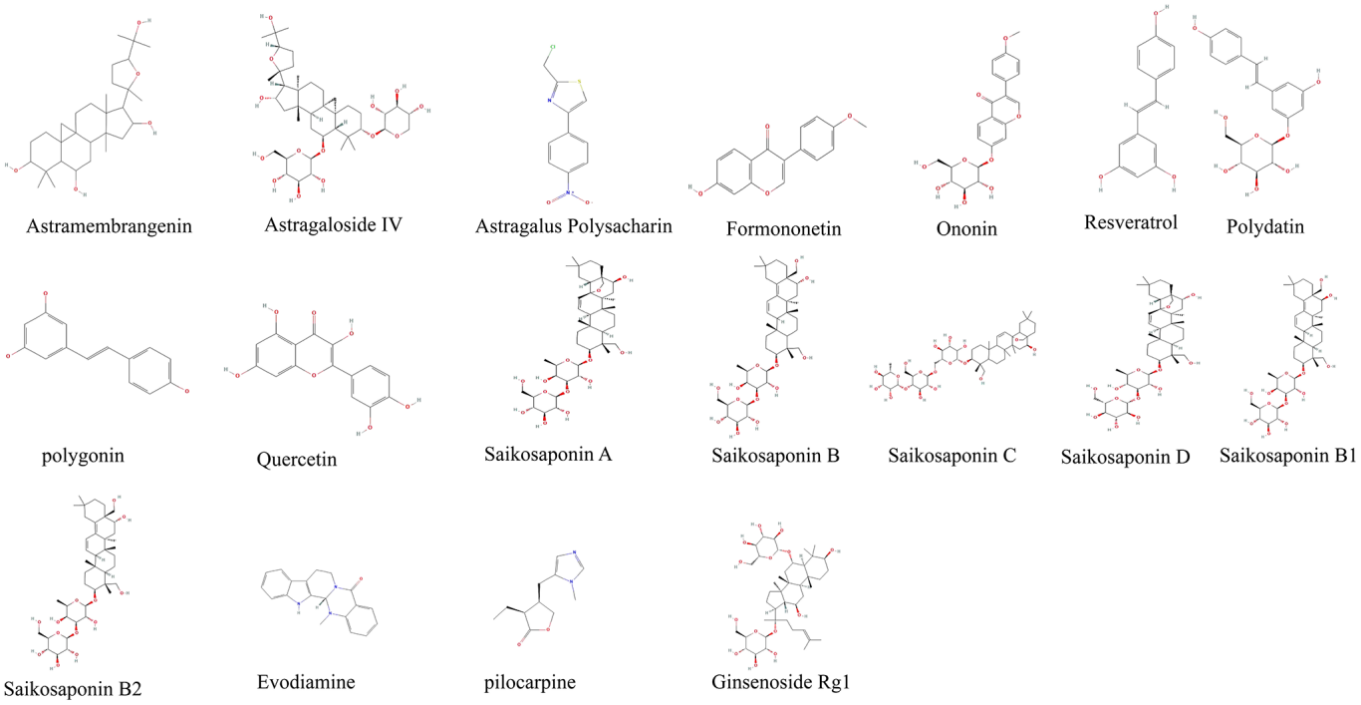
Derivatives chemical formula.

Chinese medicine was accumulated by the Chinese ancestors under specific natural and social environmental conditions. Due to the special natural conditions and the special cultural background, Chinese medicine inevitably brings its own characteristics. Both have a unique triad of characteristics: ethnicity, regionality and tradition. In Chinese medicine, the cause of ageing is due to the gradual weakening of yin and yang, and the common denominator in the treatment of disease lies in maintaining the balance of yin and yang. Among other things, in Chinese medicine theory, the spleen is the foundation of the afterlife, providing nutrients for the normal functioning of the body, while the meridians are the substances that link the tissues of the internal organs of the body [5]. If there is a dysfunction in both, it will affect the functions of the whole body, causing a dysregulation of yin and yang, which in turn leads to the onset of ageing. Radix astragali, Giant knotweed rhizome, Radix bupleuri, Fructus evodiae, Chinese ginseng are commonly used in Chinese medicine treatment to nourish the spleen and open the meridians. In this review, the above herb itself and its compounds are described for the prevention and control of cellular aging-related mechanisms (Supplementary Table 1, Figure 1).

## Radix astragali (*Astragalus membranaceus* (Fisch.) Bunge)

Radix astragali is the dried root of *Astragalus membranaceus* (Fisch.) Bunge, a member of the legume family, with the properties of strengthening the spleen, tonifying the qi, raising the yang and lifting the sockets, and benefiting the guard and consolidating the surface [6]. In traditional Chinese medicine, it is used to treat deficiencies of Qi and blood. In modern medical research, Astragalus has been found to improve immune function, enhance antioxidant, anti-radiation and anti-cancer effects, protect the cardiovascular, liver, kidney and lung, protect brain cells, diastole vascular smooth muscle, have hormone-like effects, and have antibacterial and antiviral effects [7, 8]. Cycloastragenol, Astragaloside IV, Astragalus polysaccharide are extracts from the roots of Astragalus.

Cycloastragenol is a cyclic compound that belongs to the flavonoid group and contains multiple hydroxyl groups. Due to its multiple hydroxyl groups, cycloastragenol plays an important role in combating inflammation and oxidative reactions [9].

Astragaloside IV is a naturally occurring tetraterpene that belongs to the group of flavonoid glycosides. Its chemical structure is composed of a tetracyclic triterpene alcohol backbone and a sugar group. Additionally, Astragaloside IV contains multiple hydroxyl and sugar groups, which can stabilize free radicals through reaction, thereby reducing oxidation reactions [10].

### *Astragalus membranaceus* (Fisch.) Bunge and its compounds delay cellular senescence by regulating telomerase activity

Telomeres are the genomic ends of linear chromosomes that go through successive replications of DNA to produce chromosomes with progressively shorter telomeres. When telomeres reach a critical length, they are unable to attach enough telomere capping proteins, exposing the DNA ends to the environment, activating the DNA damage response (DDR) pathway, which inhibits cell proliferation by functioning as cell cycle inhibitors p21 and p16. P21 is a protein known as cell cycle protein-dependent kinase inhibitor 1. It is a key molecule in cell cycle regulation, and its main role is to inhibit the activity of cell cycle protein-dependent kinase (CDK), thereby regulating cell growth and division [11].

c-Myc also affects the regulation of p21. As a transcription factor, c-Myc regulates the transcription of multiple genes and is involved in processes such as cell cycle regulation, cell proliferation, and cell differentiation. The aberrant expression of c-Myc is correlated with the occurrence and development of a variety of tumors, making it an important oncogene. Our study found that heat shock protein 90 kDaβ1 (HSP90B1) is a c-Myc interacting gene, which regulates p21 and consequently affects cancer cell senescence [12].

Its length is mainly regulated by telomerase, an enzyme responsible for the lengthening of telomeres in cells. Telomerase is a basic nuclear protein reverse transcriptase that adds telomeric DNA to the ends of eukaryotic chromosomes, filling in telomeres lost to DNA replication and allowing telomere repair to lengthen, which can keep telomeres from having to be lost to cell division, allowing for an increase in the number of cell divisions. Dysfunction of very short telomeres can trigger DNA damage signalling pathways [13]. Telomerase reverse transcriptase (TERT) is essential for maintaining telomerase activity, improving nuclear DNA damage and apoptosis by reducing mitochondrial reactive oxygen species (ROS) production, as well as maintaining ongoing cell division and delaying cellular senescence [8].

In a study based on rat hepatocytes, Astragalus membranaceus was found to increase the expression level of hepatic TERT and to increase telomere length [14]. In contrast, in a high glucose (HG)-based stress response in nucleus pulposus cells (NPC), Astragalus membranaceus extracts cyclogalactol and astragaloside IV were found to upregulate TERT expression, activate telomerase, and improve telomere wear, protecting NPC from HG-induced senescence [15]. Astragaloside IV, a highly purified Astragalus root extract, is often used in current studies as a telomerase activator for relevant experimental model treatments. When Astragaloside IV is treated with haploid mouse embryonic fibroblasts, it activates telomerase, causing short telomere elongation *in vivo* [16]. Furthermore, including Astragaloside IV in the normal diet of mice boosted TERT levels in the liver while also increasing glucose tolerance, osteoporosis, and skin health.

### *Astragalus membranaceus* (Fisch.) Bunge and its compounds delay cellular senescence by inhibiting oxidative stress

Oxidative stress is a key factor in the ageing process and in the onset and development of age-related diseases. Superoxide dismutase (SOD), malondialdehyde (MDA), thiobarbituric acid (TBA), 8-hydroxy-2-deoxyguanosine (8-OH-dG) and matrix metalloproteinases (MMPs) are important indicators of organ important indicators regarding oxidative stress and oxidative damage. Free radicals and peroxides are produced during biological metabolism as a result of redox reactions, and if cells are unable to scavenge free radicals and peroxides in a timely manner due to environmental stress, this leads to lipid peroxidation, protein peroxidation, and impaired defense systems caused by excess ROS, which gradually damage the structure and function of cells, eventually leading to cellular senescence [17, 18]. ROS can lead to DDR and induce cellular senescence through both mitochondrial and non-mitochondrial pathways, and, ROS-induced senescence can generate additional mitochondrial (MT) mutations and ROS, further amplifying the positive feedback loop of senescence signalling and ultimately the senescence phenotype [19]. Furthermore, ROS can stimulate autophagy, and activated autophagy in turn limits oxidative stress [20]. Free radicals are physiological by-products of metabolism that promote oxidative stress and can be converted to hydrogen peroxide and ultimately to water by a variety of antioxidant enzymes [21]. In a study based on rat hepatocytes, Astragalus membranaceus was found to inhibit oxidative stress by reducing the levels of hepatic pro-oxidants [14]. The regulatory subunit p85 and the catalytic subunit p110 of phosphoinositol 3-kinase (PI3K) form a dimer. When it binds to the growth factor receptor, it changes the protein structure of protein kinase B (Akt) and activates it, as well as activating or inhibiting the activity of a number of downstream substrates, including apoptosis-associated proteins Bad and Caspase9, regulating cell proliferation, differentiation, apoptosis, and migration. The PI3K/AKT signalling pathway is a signalling pathway associated with proliferation, differentiation and apoptosis, and is important for delaying cellular senescence and inhibiting oxidative stress and inflammatory responses [22]. Mammalian target of rapamycin (mTOR) is PI3K/Akt's downstream target, and the PI3K/AKT/mTOR pathway is crucial for controlling inflammatory responses and glycolipid metabolism [23].

In a mouse model of D-galactose (D-Gal)-induced aging, Astragalus can inhibit oxidative stress by scavenging free radicals and activating the PI3K-Akt signaling pathway, thereby delaying aging [24] In a study based on UVB (ultraviolet radiation b)-induced senescence in primary rat dermal fibroblasts, astragaloside was found to delay cellular senescence by inhibiting oxidative stress and thereby [25]. The aryl hydrocarbon receptor (AhR) is an important receptor in response to immune responses and can cause oxidative stress by promoting the production of ROS. In a study based on IS-induced oxidative stress in human renal cortical proximal tubule epithelial barrier cells, Astragaloside IV was found to inhibit oxidative stress by targeting the AhR [25].

Sirtuins (SIRTs) are a class of NAD-dependent deacetylases that are found in a wide variety of organisms, including bacteria and humans. They play a special role in the regulation of specific cellular functions by deacetylating histones and non-histones. It plays an important role in cellular senescence and biological aging by acting as a deacetylase that is directly or indirectly involved in the regulation of multiple pathways of nuclear factor-κB protein (NF-κB), P53, AMP-activated protein kinase (AMPK), mTOR, and hypoxia-inducible factor 1α (HIF-1α) through the deacetylation of certain key proteins [26, 27]. AMPK, an AMP-dependent protein kinase, is a key molecule in the regulation of biological energy metabolism and plays a critical role in the regulation of tissue energy metabolism and the immune system to delay cellular senescence [28]. In HG-induced senescence of human umbilical vein endothelial cells (HUVEC), astragaloside was found to inhibit oxidative stress through the SIRT1/AMPK pathway, thereby delaying senescence [29].

### *Astragalus membranaceus* (Fisch.) Bunge and its compounds delay cellular senescence by improving autophagy

The process of autophagy involves engulfing one's own proteins or organelles and encapsulating them into vesicles that fuse with lysosomes to form autophagic lysosomes that degrade their contents. By doing this, the cell can meet its own metabolic needs and renew some organelles that can help the organism maintain homeostatic balance. In a variety of experimental animals, dysfunctional autophagy can lead to a shortened lifespan, while enhanced or restored autophagy facilitates lifespan and healthy lifespan extension in a variety of organisms. The core process of its inhibition of senescence lies in the inhibition of mTOR or activation of AMPK [30]. Additionally, by reducing ROS production from DNA damage and encouraging the recycling of DNA repair proteins, autophagy can lessen the impacts of genomic instability and oxidative stress, as well as selectively degrading affected molecules and organelles to protect cells from ongoing DNA damage [31, 32]. Although autophagy does not restore or stop telomere attrition, studies have shown that telomere dysfunction directly stimulates autophagy and promotes precancerous cell death [33]. mTOR belongs to the PI3K family and is present in at least two protein complexes: mTOR complex 1 (MTORC1) and mTORC2 [34]. Endotoxin can down-regulate macrophage autophagy and up-regulate IL-6 via the Akt/mTOR pathway, whereas Astragalus injection reverses these phenomena and enhances autophagy by activating AMPK [35].

In a study based on the establishment of a pulmonary toxicity model based on the injection of fine airborne particulate matter2.5 (PM2.5) into rats through the trachea and PM5. 8383 into cells, Astragaloside IV was found to regulate the degradation of autophagosomes through PI3K/Akt/mTOR signalling, thereby restoring autophagic flux [36]. In a study based on the Type 2 diabetes mellitus (T2DM) rat diabetic liver injury and neonatal rat cardiomyocyte injury model, a mouse heart failure model, Astragalus polysaccharide and Astragaloside IV were found to restore normal autophagy through the AMPK/mTOR pathway [37].

### *Astragalus membranaceus* (Fisch.) Bunge and its compounds delay cellular senescence by improving mitochondrial disorders and DNA damage

Mitochondrial calcium levels, NAD^+^/NADH ratio, cardiolipin levels, mitochondrial phagocytic protein (Mieap) levels, electron transport chain function and iron metabolism are important factors affecting mitochondrial function [38]. The most prominent role of mitochondria is to generate energy for the cell to coordinate its response to environmental changes [8]. Mitochondria, a key factor in aerobic biometabolism, are the source site of oxidative stress and cellular autophagy, so mitochondrial dysfunction and reduced mitochondrial content are hallmarks of ageing and play an important role in promoting it [39, 40]. In senescent cells, decreased phosphorylation of the pyruvate dehydrogenase complex distributed in the mitochondrial matrix increases the use of pyruvate in the tricarboxylic acid cycle, leading to accelerated catabolism and redox stress, pathways that together promote cell cycle arrest [41]. Mitochondrial dysfunction is driven by loss of NAD^+^ for nuclear DNA repair, defective mitochondrial autophagy induced by DNA damage and altered expression of mtDNA polymerase which in turn affects mtDNA replication and can lead to cellular senescence [42]. A novel isoflavonoid called formononetin (FMN) was discovered in the Astragalus membranes. In a study based on HG-induced mitochondrial dysfunction in HK-2, FMN was found to reverse mitochondrial membrane depolarization and ameliorate mitochondrial damage by modulating the Sirt1/peroxisome proliFerators-activated receptor γ coactivator Lalpha (PGC-1α) pathway [43].

Mitochondrial autophagy is regulated by multiple pathways, one of which is the PINK1/Parkin pathway. In depolarized mitochondria, PTEN-induced kinase 1 (PINK1) is a molecular sensor of damaged mitochondria, triggering a signal for the initiation of mitochondrial autophagy [44]. Astragaloside effectively mitigates oxidative stress and excessive activation of mitochondrial autophagy through the PINK1/Parkin pathway to maintain mitochondrial function in Schwann Cells based on HG-induced oxidative stress [45]. Ononin is a flavonoid isolated from Astragalus root, and in LPS-treated THP-1 cell-based inflammation, Ononin ameliorates mitochondrial damage and delays the inflammatory response by triggering mitochondrial autophagy [46]. In a study based on HG-induced senescence in rat aortic endothelial cells, Astragalus polysaccharide ameliorated mitochondrial disorders by modulating mitochondrial Na^+^/Ca^2+^ [47].

### *Astragalus membranaceus* (Fisch.) Bunge and its compounds delay cellular senescence by inhibiting inflammatory responses

Increased persistent inflammation promotes the release of interleukin 6 (IL-6), interleukin 1β (IL-1β) pro-inflammatory factors and inflammatory mediators, for which upregulation can lead to immune system dysfunction, reduced cellular autophagy and thus promote aging [48]. The p53 protein is closely related to the mechanisms of aging [49]. It was found that when iron is overloaded, ROS present high levels that promote p53 acetylation, stabilize p53 by enhancing p53 binding to DNA, polarize macrophages to an M1 pro-inflammatory phenotype, promote inflammation, and in turn promote cellular senescence [50]. SIRT1 performs the function of a histone deacetylase on p53, which operates to deacetylate p53 so that inflammation is inhibited and thus slows down ageing [51]. In a study based on a D-Gal-induced senescence model of HK-2 cells, Astragaloside IV was found to alleviate the inflammatory response through the SITR1-p53 pathway, thereby delaying cellular senescence [52]. In studies based on IL-3β-induced RSC-1 inflammatory response and ovalbumin-induced asthma model in mice, Astragalus polysaccharide A and Astragaloside IV exerted anti-inflammatory effects to alleviate the inflammatory response by inhibiting PI3K/AKT/mTOR autophagic pathway [53, 54]. Toll-like receptors (TLRs), which recognize various molecular patterns associated with pathogens, are crucial for the innate immune response. They serve a crucial function in inflammation, immune cell regulation, survival, and proliferation and are the first line of defense against pathogen invasion. They also activate NF-κB [55]. In contrast, NF-κB is a key regulator of natural and acquired immune responses, with functions in promoting cell proliferation, inhibiting apoptosis, promoting cell migration and invasion, and stimulating angiogenesis and metastasis. As an upstream gene for a variety of inflammatory factors, activation of NF-κB promotes the release of a variety of pro-inflammatory factors (IL1, IL6 and IL8), which in turn promotes cellular senescence [56]. In a study on the construction of a mouse model of idiopathic pulmonary fibrosis based on bleomycin (BLM), Astragalus polysaccharide was found to attenuate the inflammatory response by inhibiting the TLR4/NF-κB signaling pathway [57].

### *Astragalus membranaceus* (Fisch.) Bunge and its compounds delay cellular senescence by regulating metabolism

The broad name for the orderly succession of chemical events that take place in living things to sustain life is metabolism, sometimes known as metabolism. These reactionary mechanisms enable organisms to develop and reproduce, preserve their structure, and react to their environment. Catabolism, which uses energy to break down big molecules, and anabolism, which uses energy to synthesise parts of the cell like proteins and nucleic acids, are the two categories into which metabolism is typically classified. An organism's metabolism can be conceived of as the continuous interchange of matter and energy within it; when this exchange stops, the organism's structure disintegrates.

Glucose and lipid metabolism are by far the largest factors affecting cellular senescence, and disorders of glucolipid metabolism can lead to inflammation, intestinal flora disorders, and mitochondrial dysfunction [58–60]. While calorie restriction (CR), which decreases telomere wear, boosts antioxidant capacity, and lowers ROS production, is the most efficient regulated metabolic strategy to delay aging. Other studies have demonstrated that CR can promote mitochondrial proliferation by activating the PGC-1 in mitochondria to promote mitochondrial proliferation and thus mitigate oxidative damage by generating energy. CR can also promote ATP synthesis by balancing respiratory movements [8].

T2DM is a systemic metabolic disorder characterized by insulin deficiency and insulin resistance. In a study based on a T2DM mouse model, astragaloside and astragaloside polysaccharide were found to improve the disruption of glucolipid metabolism in T2DM mice, and astragaloside polysaccharide protected the intestinal barrier by suppressing intestinal inflammation and oxidative stress levels, inhibiting the potential intestinal pathogen Shigella, and promoting the growth of beneficial bacteria ectopic and lactobacilli [61, 62]. Sweet taste receptors (STR) play an important role in glucose metabolism and can act as glucose sensors, expressed in intestinal sweet cells and intestinal cell populations involved in sugar transport *in vivo*, with downstream targets of taste receptor family 1 member 2 (T1R2), α-gustadusin (Gα) and transient receptor potential cation channel subfamily member 5 (TRPM5) [63]. In a study based on T2DM rats, Astragalus polysaccharides were found to promote glucose transport and lipogenesis through activation of the STRs pathway to promote glucose metabolism [64].

### *Astragalus membranaceus* (Fisch.) Bunge and its compounds delay cellular senescence by improving intestinal flora dysbiosis

In general terms, the microorganisms in the gut are divided into three main groups: beneficial, harmful and neutral bacteria. The microorganisms in the gut are divided into several phyla, namely Firmicutes, Bacteroidetes, Actinobacteria, Proteobacteria, Verrucomicrobia, etc., of which Firmicutes and Bacteroidetes dominate in number. The dominant phyla are Firmicutes and Bacteroidetes [65]. Intestinal motility and diet structure can lead to changes in the distribution and structure of the intestinal flora [66]. Ageing is associated with intestinal flora. If the intestinal flora is disturbed, it can trigger oxidative stress, inflammatory responses and consequently cellular senescence [67]. In the study, it was found that the associated increase in gut flora, affects cellular senescence, as well as affecting oxidative stress and mitochondrial dysfunction in cells [68]. RSS produced by the intestinal flora enhances the antioxidant capacity of the host [69]. In contrast, intestinal flora maintains intestinal homeostasis through the release of short-chain fatty acids (SCFAs). Following the fermentation of dietary fiber and resistant starch, certain colonic anaerobic bacteria create SCFAs, which mostly consist of acetic acid, propionic acid, and butyric acid. The formation of SCFAs is the result of complex interactions between diet and intestinal flora in the intestinal luminal environment and has an important role in the immune, metabolic and endocrine aspects of the body [68, 70]. The production of SCFAs is a fermentation reaction that occurs in the lumen of the large intestine, and the main bacteria that produce SCFAs are anaerobic bacilli, bifidobacteria, eubacteria, streptococci and lactobacilli [71].

In a study based on a high-fat diet-induced non-alcoholic fatty liver disease (NAFLD) rat model, Astragalus polysaccharides ameliorated liver inflammation and lipid accumulation in NAFLD by modulating gut microbiota signalling pathways [72]. In a BLM (BLM)-based mouse model of idiopathic pulmonary fibrosis (IPF), Astragalus polysaccharides were found to attenuate the inflammatory response by inhibiting the TLR4/NF-κB signalling pathway and to balance the gut microbiota by regulating metabolic pathways [57]. In a study based on a mouse model of T2DM, Astragalus polysaccharide was found to improve disorders of glucolipid metabolism in T2DM mice and protect the intestinal barrier by suppressing levels of intestinal inflammation and oxidative stress, inhibiting the potentially pathogenic intestinal bacteria Shigella and promoting the growth of the beneficial bacteria Fusobacterium and Lactobacillus [57]. In a mouse model of cholesterol gallstones, Astragalus polysaccharide enhanced bile acid synthesis and improved intestinal microbiota, increasing the relative abundance of the phylum Bacteroides [73].

## Giant knotweed rhizome (*Reynoutria japonica* Houtt.)

The Giant knotweed rhizome of the plant *Reynoutria japonica* Houtt. is used in traditional Chinese medicine to dispel wind, promote dampness, break up blood stasis and clear the channels [6]. In modern research, it is important in combating cellular ageing and in orthopaedic and gynaecological diseases [74, 75]. Resveratrol, Polydatin, Polysaccharide and Thujaplicins are all extracts of the Chinese wildflower. Resveratrol is a natural product belonging to the class of compounds known as flavonoids. Its chemical structure comprises two benzene rings and a propenyl side chain. The benzene ring structure and propenyl side chain enable it to effectively stabilize free radicals, consequently mitigating cellular damage caused by them [76]. Polydatin is a naturally occurring polyphenol compound. Polydatin's antioxidant properties are mainly attributed to the benzene ring and glucose moiety in its chemical structure. The hydroxyl group in the benzene ring structure has a powerful free radical scavenging ability that neutralizes and stabilizes free radicals, thereby reducing free radical damage to cells and tissues. In addition, the presence of the glucose moiety makes Polydatin more water-soluble for better antioxidant effects in the body [77].

Thujaplicins are natural compounds that belong to the saponin group. Its chemical structure is composed of a sugar group and a saponin backbone. The hydroxyl group in the sugar group and the unsaturated bond in the fatty acid group of Thujaplicins can supply hydrogen to participate in the neutralization reaction of free radicals, thereby inhibiting the oxidation reaction. Additionally, saponins can inhibit metal-catalyzed oxidation reactions by binding to metal ions [78].

### *Reynoutria japonica* Houtt. and its compounds delay cellular senescence by enhancing telomerase activity

p53 is a common human tumour suppressor protein and transcription factor involved in the regulation of genome integrity, cell cycle arrest, apoptosis, and autophagy [79]. Endogenous wild-type p53 promotes senescence by inhibiting TERT, which in turn down-regulates telomerase activity [80]. Resveratrol, a tiger cane extract, was found to increase telomerase activity by reducing p53 levels in a study based on mechanical overload-induced senescence in human myeloid cells [81]. Resveratrol, the main extract of Eryngium tigrinum, has been found to delay cellular senescence by activating the PI3K-Akt signaling pathway and increasing telomerase activity in studies based on naturally aging endothelial progenitor cells (EPCs) [82].

### *Reynoutria japonica* Houtt. and its compounds delay cellular senescence by inhibiting oxidative stress

In angiotensin II (AngII)-based induced vascular smooth muscle cell senescence, resveratrol can increase the expression of antioxidants and inhibit oxidative stress by activating the AMPK-sirt1 signaling pathway, thereby delaying aging [83]. In addition, resveratrol inhibits bone marrow stromal stem cells via the mitogen-activated protein kinase (MAPK) pathway, an upstream activation signal for Akt and AMPK, reducing senescence-related phenotypes and oxidative stress [28, 84]. The MAPK pathway includes the classical MAPK pathway, the c-Jun amino-terminal kinase (JNK)/p38 mitogen-activated protein kinase (p38 MAPK) pathway and extracellular signal-regulated kinase 5 (ERK5). Among these, ERK primarily controls cell growth and differentiation whereas JNK and p38 mostly control inflammation, apoptosis, and proliferation [85]. Peroxisome proliferator-activated receptor γ (PPARγ) is a key regulator of adipogenesis and adipose tissue development and has an important role in the inhibition of oxidative stress due to lipid peroxidation [86, 87]. In studies based on lipopolysaccharide-induced senescence in endothelial progenitor cells, resveratrol inhibits oxidative stress and thus delays senescence through the PPAR-γ pathway [88].

NRF2 has been identified as a crucial transcription factor that mediates oxidant defense and improves cell viability in numerous tissues [89]. NRF2 deficiency results in delayed proliferation of maternal hepatocytes with associated dysregulation of cell cycle protein activation [90]. And NRF2 plays an important role in maintaining normal oxygen dynamic homeostasis and restoring normal dynamic homeostasis after oxidative damage. The glutathione pathway is a key pathway for the restoration of redox homeostasis, and when glutathione (GSH) levels are reduced, this can lead to disruption of the cellular antioxidant system and the inability to eliminate ROS, leading to a build-up of ROS [91]. The interaction of NRF2 with NF-κB is essential for integrating the inflammatory response. In the presence of inflammation, NF-B is upregulated and consequently its target NRF2 transcription is reduced, allowing for a sustained increase in the production of pro-inflammatory cytokines that underlie these diseases. The removal of NRF2 increases the activity and cytokine production of NF-B, which can positively or negatively regulate the transcriptional activity of NRF2 [92].

HIF-1α expression is increased during hypoxia and can reduce ROS levels during hypoxia through multiple pathways, including direct targeting of mitochondria to prevent oxidative stress [93]. Nicotinamide adenine dinucleotide phosphate (NADPH) oxidase (NOX) is composed of a membrane subunit (NOX1, NOX2, NOX3, NOX4 or NOX5) and a catalytic subunit (p22phox, p47phox, p67phox) and is one of the main sources of ROS production in cells [94]. Nuclear factor (erythroid-derived 2) like 2 (NRF2) is a member of the basic leucine transcription factor family, which is implicated in redox regulation, protein stabilization, DNA repair, and prevention of apoptosis [95]. When NOX levels are increased, HIF-1α can be activated [96]. In HG-induced glomerular podocyte and human retinal epithelial cell injury, Polydatin and Thujaplicins can modulate the NRF2 signalling pathway and alleviate oxidative stress by inhibiting HIF-1α/NOX4 [97, 98]. In studies based on D-gal-induced cardiac senescence in mice and renal senescence in rats, Thujaplicins attenuated cardiac and renal senescence by inhibiting oxidative stress [99, 100]. In a study based on ethanol-induced liver injury in mice, aqueous extracts of C. tigrinus inhibited hepatic oxidative stress by modulating NRF2 [101].

### *Reynoutria japonica* Houtt. and its compounds delay cellular senescence by improving autophagy

In a study based on palmitic acid-induced muscle cell senescence, resveratrol was found to inhibit cellular senescence by restoring autophagic flux [102]. Neurogenic locus notch homolog protein 1 (Notch1) is a transmembrane receptor that can be involved in apoptotic activity by regulating autophagy [103]. In studies based on cigarette smoke-treated endothelial cells, resveratrol reversed the decrease in mTOR expression caused by cigarette smoke through Notch1 signalling, thereby promoting autophagy (Zong DD, Liu XM, Li JH, Ouyang RY, Long YJ, Chen P, Chen Y. Resveratrol attenuates cigarette smoke induced endothelial apoptosis by activating Notch1 signaling mediated autophagy. Respir Res. 2021 Jan 19; 22(1):22.).

Inflammatory vesicles are cytoplasmic multiprotein complexes that primarily mediate the host immune response to microbial infection and cellular damage. Pattern recognition receptor (PRR) is one of the components of inflammatory vesicles, and NLRP3 belongs to the family of NOD-like receptor proteins (NLRs) among the PRRs that form inflammatory vesicles. NLRP3 inflammatory vesicles are activated by two main signals: the first initiation signal is provided by microbial or endogenous molecules that induce NLRP3 inflammatory vesicle expression by activating NF-κB; the second activation signal is triggered by adenine nucleoside triphosphate, pore-forming toxins, viral RNA or particulate matter (DING Yang (丁杨), HU Rong. Research Progress in Mechanisms of NLRP3 Inflammasome Activation and Regulation[J]. Progress in Pharmaceutical Sciences, 2018, 42(4):294-302 (in Chinese). When the environment is hypoxic, it reduces the binding of NLRP3 and mTOR and activates autophagy to improve the inflammatory response (Cosin-Roger, J., Simmen, S., Melhem, H., Atrott, K., Frey-Wagner, I., Hausmann, M., de Vallière, C., Spalinger, M.R., Spielmann, P., Wenger, R.H., Zeitz, J., Vavricka, S.R., Rogler, G., Ruiz, P.A., 2017. Hypoxia ameliorates intestinal inflammation through NLRP3/mTOR downregulation and autophagy activation. Nat. Commun. 8, 98. https://doi.org/10.1038/s41467-017-00213-3). Polydatin is a bioactive component of thuja, a natural precursor of resveratrol. Polydatin prevented the activation of NLRP3 inflammatory vesicles, restricted the production of inflammatory cytokines, and promoted autophagy via the NLRP3/mTOR pathway in atherosclerotic lesions generated in mice based on feeding high fat (Zhang X, Wang Z, Li X, Chen J, Yu Z, Li X, Sun C, Hu L, Wu M, Liu L. Polydatin protects against atherosclerosis by activating autophagy and inhibiting pyroptosis mediated by the NLRP3 inflammasome. J Ethnopharmacol. 2023 Jun 12; 309:116304).

### *Reynoutria japonica* Houtt. and its compounds delay cellular senescence by ameliorating mitochondrial disorders and DNA damage

In studies based on human embryonic lung fibroblasts and human peritoneal mesothelial cells (HPMC), resveratrol treatment was found to delay cellular senescence by attenuating oxidative DNA damage, mitochondrial dysfunction [104, 105]. In studies based on hydrogen peroxide-induced senescence in HUVEC and human nucleus pulposus cells (NPC), resveratrol was found to attenuate mitochondrial dysfunction through autophagy, thereby delaying cellular senescence [106, 107]. In human nasal epithelial cell stress-based and mouse allergic rhinitis models, Polydatin ameliorated mitochondrial damage through PINK1-Parkin-mediated mitochondrial autophagy and exerted a protective effect against allergic rhinitis by inhibiting NLRP3 inflammatory vesicles [108].

### *Reynoutria japonica* Houtt. and its compounds delay cellular senescence by inhibiting inflammatory responses

miRNAs are a class of endogenous non-coding small molecule RNAs involved in the regulation of metabolism, inflammatory responses, mitochondrial disorders, oxidative stress responses and other mechanisms related to cellular senescence [109]. In a study based on cigarette smoke extract (CSE)-induced senescence in human airway epithelial cells (BEAS-2B), resveratrol was found to delay cellular senescence by modulating the miR-2a/SIRT1/NF-κB pathway [110]. In a study based on inflammatory cytokine-induced inflammatory responses in nucleus pulposus (NP) cells, resveratrol was found to increase telomerase activity and reduce the expression of inflammatory proteins, thereby delaying cellular senescence [111]. In studies based on an anterior cruciate ligament transection-induced osteoarthritis rat model and IL-1β treatment of rat chondrocytes, Polydatin attenuated inflammation by inhibiting the NF-κB signalling pathway *in vitro* [112].

The signal transducer and activator of transcription protein signal transduction and activator of transcription (STAT) is a unique family of proteins that bind to DNA and have an important role in cancer progression and inflammatory responses [113]. In a study based on dextran sodium sulfate (DSS) and 2,4,6-trinitrobenzenesulfonic acid (TNBS)-induced colitis in mice, polydatin inhibited Th3 cell differentiation and attenuated the inflammatory response by directly inhibiting signal transduction and activator of transcription 17 (STAT17) [114].

### *Reynoutria japonica* Houtt. and its compounds delay cellular senescence by regulating metabolism

In studies based on HG and hyperinsulin-induced inflammation in adipocytes, thuja polysaccharide could regulate glucolipid metabolism and alleviate the inflammatory response by promoting NRF2 expression [115]. In Oxidized Low Density Lipoprotein (ox-LDL)-based stimulation of HUVEC, polydatin reversed ox-LDL-induced apoptosis and inhibition of lipid accumulation and angiogenesis in HUVEC cells by modulating the miR-26a-5p/BID axis to ameliorate dysfunctional lipid metabolism [116]. Interleukin-1 receptor-associated kinase 3 (IRAK3) is an important negative regulator of TLR-mediated cell signaling that inhibits activation of the NF-κB signaling pathway and has an important role in the inflammatory response [117].

In palmitate-based treatment of INS-1 insulinoma cells and diabetic mouse models, polydatin alleviated dyslipidemia and reduced insulin resistance by enhancing insulin secretion and expression of diabetes-related genes [118].

Similarly, also in studies of the myotubular insulin resistance model induced by palmitic acid, Thujaplicins ameliorated glucose metabolism disorders and ameliorated HG-induced inflammatory responses by modulating miR-6-340p/IRAK3 [119]. Scholesterol regulatory element binding proteins (SREBPs) and peroxisome proliferator-activated receptor alpha (PPARα) [120]. It has an important role in lipid metabolism and can act as a signalling factor for normal or abnormal lipid metabolism [120, 121]. In studies based on hyperlipidemic and obese mouse models, Thujaplicins reduced the expression levels of SREBPs and PPARα and exerted regulatory lipid metabolism and anti-inflammatory effects through activation of the AMPK signaling pathway [122, 123]. In studies based on T2DM mice, Thujaplicins and aqueous extracts of C. tigris reduced blood glucose levels and blood lipid levels in mice, and Thujaplicins also repaired pancreatic β-cells to improve disorders of glucolipid metabolism [124, 125].

### *Reynoutria japonica* Houtt. and its compounds delay cellular senescence by improving intestinal flora dysbiosis

*Reynoutria japonica* Houtt. has an important role in improving the dysbiosis of intestinal flora, but no more detailed regulatory mechanism has been proposed in the current study. In a study of an immunosuppressed mouse model based on cyclophosphamide, ethanolic extracts of C. tigrinus restored the gut microbial community disturbed by cyclophosphamide [126]. In a study based on a high-fat diet-induced obesity in mice, resveratrol treatment significantly modulated the composition and metabolic function of the intestinal flora and exerted an anti-obesity effect [127]. Resveratrol was discovered to lower inflammatory responses and regulate the expression of target genes related to lipid metabolism by regulating intestinal microecological balance and encouraging the growth of probiotic bacteria in a study of rats with metabolic syndrome (MS) induced by a high-fat, high-salt, high-sugar diet [128]. In a study of mice fed a high-fat diet, resveratrol was found to alter the intestinal microbial composition, reducing the proportion of thick-walled bacteria that could be pro-inflammatory and the ratio of thick-walled bacteria to anaphylactic bacteria [129]. In cyclophosphamide-based mouse studies, ethanol extracts of C. tigrinus restored disturbed intestinal flora [126].

## Radix bupleuri (*Bupleurum scorzonerifolium*.)

Radix bupleuri is the dried root of *Bupleurum scorzonerifolium*. family Umbelliferae, which has the effect of reconciling the exterior and interior, draining the liver and Qi, and raising Yang [6]. Modern research has shown that its antidepressant, anti-inflammatory, immunomodulatory, hepatoprotective, and anti-aging effects are important in the prevention and control of cellular aging [130]. Quercetin, Caihu polysaccharide and Caihu saponin are the main active ingredients of Caihu.

Quercetin is a naturally occurring flavonoid that possesses multiple phenolic hydroxyl groups and double bonds in its chemical structure. These features enable it to effectively trap and neutralize free radicals, thus reducing oxidative stress and cellular damage [131].

Caihu saponin is a natural compound extracted from *Bupleurum scorzonerifolium*. It is a triterpenoid. Caihu saponin's chemical structure consists of a benzene ring and a hydroxyl group. With its high electron density, it can effectively absorb and neutralize free radicals. Consequently, it mitigates oxidation reactions induced by free radicals [132].

### *Bupleurum scorzonerifolium*. and its compounds delay cellular senescence by regulating telomerase activity

Quercetin is one of the active ingredients of radix bupleuri. In a study of Oxidized Low Density Lipoprotein (ox-LDL)-induced senescence in human aortic endothelial cells (HAEC), quercetin was found to reduce the increase in telomerase activity in endothelial cells caused by ox-LDL In a study of human aortic endothelial cell (HAEC) senescence, quercetin was found to reduce the increase in telomerase activity in endothelial cells caused by ox-LDL, thereby delaying senescence [133].

### *Bupleurum scorzonerifolium*. and its compounds delay cellular senescence by inhibiting oxidative stress

In H_2_O_2_-induced vascular smooth muscle cell (VSMC)-based senescence, quercetin inhibits oxidative stress by activating AMPK, which in turn inhibits VSMC senescence [134]. Chai Hu polysaccharide, the main active ingredient in Chai Hu, has significant *in vitro* antioxidant effects, and it can significantly delay hydrogen peroxide (H_2_O_2_)-induced senescence in mouse lung epithelial cells [135]. Chaihu saponin D can regulate PI3K/NRF2, p38-MAPK signaling pathway and thus inhibit senescence in each cell by reducing oxidation levels in studies based on neuronal cell inflammation, cardiomyocyte inflammation, and hose cell inflammation [136–138].

### *Bupleurum scorzonerifolium*. and its compounds delay cellular senescence by improving autophagy

In a study based on a tert-butyl hydroperoxide (TBHP)-induced oxidative stress model of NPC, quercetin was found to enhance autophagy through modulation of the p38MAPK/mTOR signaling pathway to protect NPC from apoptosis and thus delay cellular senescence [139]. Caihu saponin D was discovered to reduce the release of inflammatory factors in mice in a study based on carbon tetrachloride-induced liver fibrosis in mice through enhancing autophagy [140].

### *Bupleurum scorzonerifolium*. and its compounds delay cellular senescence by ameliorating mitochondrial disorders and DNA damage

The mitochondrial membrane potential of aged HDL fibers treated with quercetin was found to be restored to levels comparable to those of young HDL fibers in a study using human dermal fibroblasts (HDF), indicating that quercetin reversed age-related changes in mitochondrial membrane potential and improved mitochondrial dysfunction [27]. In a rat neuronal cell-based study, Caihu saponin D ameliorated mitochondrial damage by modulating mitochondrial translocation and chromosome-dependent pathways [141].

### *Bupleurum scorzonerifolium*. and its compounds delay cellular senescence by inhibiting inflammatory responses

Ethanolic extracts of C. tibetica reduced inflammation by blocking the NF-κB signaling pathway, which in turn reduced inflammation in tests using lipopolysaccharide to produce inflammation and neuritis in rats [142]. In IL-1β-based treatment of senescent nasopharyngeal carcinoma cells, quercetin was found to retard senescence by increasing NRF2 levels and impeding NF-κB signaling [143]. In studies based on cigarette smoke and lipopolysaccharide-induced lung inflammation in mice and endometritis in mice, respectively, Chai Hu saponin A inhibited the inflammatory response through the NRF2/NF-κB pathway [144, 145]. In lipopolysaccharide-induced acute lung Injury-based mice, chaihu saponin A and chaihu saponin b1/b2, inhibited the inflammatory response by suppressing the NF-κB/TLR4 signalling pathway [146]. Similarly, in mice with lipopolysaccharide-based induced macrophage inflammation and acute liver injury, Chaihu saponin B reduced Sirt6 expression levels and thus inflammation levels by inhibiting NF-κB signaling [147, 148].

In response to osmotic pressure gradients, the selective water channel protein aquaporin 1 (AQP1), which is confined to glial cells in the human peripheral nervous system, makes it easier for water to penetrate cell membranes [149]. AQP1 activates ras homolog family member A (RhoA), which is expressed at the plasma membrane, and the RhoA/Rho-related protein kinase (ROCK) signalling pathway plays an important role in glycolipid metabolism [150, 151]. In studies based on depressed mice and rats with streptozotocin and high-fat diet-induced diabetes, Chai Hu saponin C inhibited inflammatory responses by suppressing inflammatory factor secretion and by modulating the AQP1/RhoA/ROCK signaling pathway [149, 152]. In studies based on LPS-induced neuroinflammation/microglia activation in mice and carbon tetrachloride-induced liver fibrosis in mice, chaihu saponin D suppressed inflammatory responses by regulating the NF-κB/TLR4 signaling pathway, and by inhibiting NLRP3 inflammatory vesicle expression [153–156].

### *Bupleurum scorzonerifolium*. and its compounds slow down cellular senescence by regulating metabolism

In studies based on a high-fat, HG diet-induced disorder of glucolipid metabolism model, Chai Hu total saponin, Chai Hu saponin A, and Chai Hu saponin D all reduced triacylglycerol (TG) or Serum total cholesterol (TC) levels by enhancing metabolism [157, 158]. Among them, Caihuosaponin A and Caihuosaponin D could affect metabolism by regulating the PPAR pathway. In addition, in a study of lipopolysaccharide/galactosamine-induced acute liver injury in mice, chaihu saponin b2 enhanced metabolism by upregulating Sirt6, and increasing the expression of Na^+^-K^+^-ATPase, Ca^2+-^Mg^2+^-ATPase [148].

### *Bupleurum scorzonerifolium*. and its compounds delay cellular senescence by improving dysbiosis of the intestinal flora

In a study based on sodium taurocholic acid-induced severe acute pancreatitis in rats, Caihu saponin A could regulate intestinal flora distribution through activation of the NRF2 pathway [159]. In contrast, in a study of dextran sodium sulfate-induced ulcerative colitis in mice, chaihu saponin D reduced the expression of IL-1β, NF-κB inflammatory factors, and increased the levels of enteric beneficial bacteria while decreasing the levels of harmful bacteria [160].

## Fructus evodiae (*Evodia rutaecarpa* (Juss.) Benth)

Fructus evodiae is the dried fruit of the plant *Evodia rutaecarpa (Juss.) Benth*. It has the effect of regulating Qi, warming the middle and relieving pain [6]. In modern research, Cornus officinalis has been shown to have good anti-inflammatory, anti-tumor, antioxidant and anti-aging effects, which are important in the prevention and control of cellular aging [161]. Evodiamine and rutin are the primary active compounds found in *Evodia rutaecarpa* (Juss.) Benth.

Evodiamine is a plant compound that exhibits a broad spectrum of biological activities. It possesses a chemical structure comprising multiple aromatic rings and hydroxyl groups, which confer antioxidant activity and the capacity to scavenge free radicals, consequently mitigating oxidative reactions induced by free radicals [162]. Rutin is a naturally occurring flavonoid that contains a sugar group. The hydroxyl group in the sugar group and the unsaturated bond in the fatty acid group provide hydrogen, which is involved in neutralizing free radicals and inhibiting the oxidation reaction [163].

### *Evodia rutaecarpa* (Juss.) Benth and its compounds delay cellular senescence by inhibiting oxidative stress

Phosphoglycerate kinase 1 (PGK1) is an important enzyme for ATP production and plays an important role in metabolism [164]. When PGK1 activation is inhibited, NRF2 expression can be promoted [165].

In a study based on traumatic brain injury (TBI) caused by controlled cortical shock, evodiamine extract evodiamine was found to inhibit oxidative stress responses by targeting PGK1/NRF2 signaling [166]. In a study based on HG-induced endothelial cell senescence, evodia extract rutine can exert antioxidant activity by upregulating SIRT1 expression, thereby delaying cellular senescence [167]. In addition, rutine can upregulate NRF2 expression and inhibit PIK3 expression in mice with ethanol-induced gastric mucosal injury, inflammatory bowel disease, and cerebral ischemia-reperfusion rats. This inhibits oxidative stress and delays the inflammatory response [168–170].

JNK is a member of the mitogen-activated protein kinase family, which has the function of phosphorylating and activating the protein cJun, which reduces fatty acid oxidation, ketone production, and promotes steatosis during diet-induced obesity, which can eventually lead to a series of pathological changes [171]. In studies based on renal ischemia-reperfusion mice, rutine can reduce oxidative stress through the p38-MAPK and cJun-MAPK signaling pathways [172].

Furthermore, in studies based on HG and doxorubicin-induced cardiomyocytes and cardiotoxic mice, respectively, rutine can reduce oxidative stress and inhibit inflammation through the MAPK signaling pathway and increase NRF2 expression levels [173, 174].

### *Evodia rutaecarpa* (Juss.) Benth and its compounds delay cellular senescence by improving autophagy

There is less research on the regulation of autophagy and its compounds in regulating autophagy and thus delaying cellular aging, mainly related to brain diseases and colon diseases. In astrocytes-based studies, evodiamine enhances autophagy by regulating the JNK/p38 MAPK signaling pathway [175]. In the study of dextran sulfate-induced colitis in mice, evodiamine can inhibit the inflammatory response by inhibiting the expression levels of NF-κB and NLRP3, and can also upregulate the number of autophagosomes, thereby enhancing autophagy [176].

### *Evodia rutaecarpa* (Juss.) Benth and its compounds delay cellular aging by improving mitochondrial disorders and DNA damage

In studies of hydrogen peroxide-stimulated mouse hepatocytes, rutine can improve DNA damage by regulating the JNK/p38 MAPK signaling pathway and the PI3K/Akt signaling pathway [177].

In addition, in studies of endothelial inflammation and DNA damage in human umbilical vascular endothelial cells based on 25-hydroxycholesterol, tumor necrosis factor α(TNFα), and tert-butyl hydrogen peroxide-stimulated hepatocytes, rutine can improve DNA damage by upregulating NRF2 expression levels and inhibiting the increase of oxide levels [178, 179].

TGFβ is the main regulator of extracellular matrix accumulation and a potential key driver of hepatitis to liver fibrosis, TGF-β1 is the most effective cytokine to promote liver fibrosis, can inhibit the proliferation of hepatocytes, stimulate the activation of hepatic stellate cells (HSCs), promote the production of ECM, and regulate apoptosis of hepatocytes [180]. In a mouse study of paracetamol-induced acute liver injury, rutine upregulated NRF2 expression levels, thereby hepatic inflammatory response, and improved mitochondrial disorders in mouse hepatocytes [181].

### *Evodia rutaecarpa* (Juss.) Benth and its compounds delay cellular senescence by inhibiting the inflammatory response

In a colitis-based study in mice, evodiamine inhibited the inflammatory response by inhibiting NF-κB and NLRP3 inflammasomes, as well as enhancing autophagy and altering intestinal flora structure [176, 182]. In Al(OH)_3_ mixture-induced asthmatic mice and lipopolysaccharide-induced HUVEC injury, evodiamine can delay the inflammatory response by inhibiting the NF-κB/TLR-4 signaling pathway [183, 184]. In studies based on the stimulation of macrophage lipids and mouse mammary epithelial cells, respectively, evodiamine and rutine can inhibit the occurrence of inflammatory responses by inhibiting the p38/JNK-MAPK signaling pathway [185, 186].

In lipopolysaccharide-induced injury in rat kidney cells and mouse microglia, evodiamine can inhibit the inflammatory response by inhibiting NF-κB and increasing NRF2 expression levels [187, 188]. In addition, also in lipopolysaccharide-induced human NPC injury, evodiamine can also inhibit the inflammatory response by upregulating SIRT1 expression levels [189].

Apelin (APJendogenousligand) is an endogenous ligand of angiotensin domain type 1 receptor-associated protein angiotensin receptor-like 1. In a mouse study based on lipopolysaccharide-induced lung inflammation and fibrosis, evodiamine was found to reduce inflammatory response and fibrosis by activating the apelin pathway [190].

In studies of rat pancreatic exocrine tumor cells treated with azurin and LPS-induced acute pancreatitis in mice, rutine inhibits inflammatory response via the NF-κB/MAPK signaling pathway [191]. In the study of acetaminophen-induced hepatotoxicity in mice, rutine inhibited the occurrence of inflammatory response by increasing NRF2 expression levels [181].

### *Evodia rutaecarpa* (Juss.) Benth and its compounds delay cellular aging by regulating metabolism

mTOR plays an important role not only in autophagy, but also in lipid metabolism [192]. In a high-fat diet-induced obesity/diabetes mouse model, evodiamine can mediate metabolism via the AMPK/mTOR signaling pathway [193]. In a study of T2DM rats treated with a high-fat diet combined with streptozotocin (STZ), evodiamine was found to reduce blood glucose in T2DM rats and improve hyperglycemia and hyperlipidemia in T2DM rats [194].

In studies based on streptozotocin-induced hyperlipidemia/hyperglycemia rats and mouse models of T2DM, rutine was found to improve the model's glycolipid metabolism by regulating the PI3K/Akt signaling pathway in the liver and activating the AMPK signaling pathway [195]. In studies based on high-fat diet-induced hyperlipidemia/obesity mice and rats with myocardial infarction, evodiamine was found to significantly improve metabolic lipid profiles by promoting the PPARγ signaling pathway, and rutine by reducing PPARα expression levels, which in turn reduced liver lipid accumulation [196, 197].

### *Evodia rutaecarpa* (Juss.) Benth and its compounds delay cellular senescence by improving intestinal dysbacteriosis

In studies based on carbon tetrachloride-induced liver fibrosis mice and pyrimidinmethane -induced colitis mice, evodiamine (EVO) was found to promote bacterial enrichment that can produce SCFAs, reduce the level of pro-inflammatory bacteria, and thus inhibit the occurrence of inflammatory response [182, 198, 199].

## Chinese ginseng (*Panax ginseng* C. A. Meyer)

Chinese ginseng is the dried root of *Panax ginseng* C. A. Meyer. In traditional Chinese medical theory, it is a great tonic for vital energy, restores the pulse and fixes the detachment, tonifies the spleen and benefits the lung, generates fluid and calms the mind [6]. Modern studies have found that ginseng has pharmacological effects such as stimulating nerve centers, anti-tumor, protecting cardiovascular and cerebrovascular diseases, improving immunity, delaying aging, lowering blood lipids and anti-fatigue [200]. Ginsenoside Rg1 and ginseng polysaccharide are the primary active constituents of Panax ginseng C. A. Meyer.

Ginsenoside Rg1 is a natural product with a tetracyclic structure and multiple sugar groups in its chemical composition. The functional groups in the chemical structure of Ginsenoside Rg1, such as the tetracyclic structure and sugar groups, have the ability to react with oxidizing substances like free radicals. This allows them to scavenge free radicals and reduce the damage caused by oxidative stress [201].

### *Panax ginseng* C.A. Meyer and its compounds delay cellular senescence by regulating telomerase activity

p16^INK4a^-Rb and pl9Arf-p53-p21Cipl are telomere-independent and telomere-dependent signaling pathways, respectively. The two key molecular signaling pathways of HSC aging, either signaling pathway activation can induce HSC aging, when the key regulatory factors involved in these pathways are altered, cells will undergo aging or bypass the aging process to continue to proliferate, but there is extensive multi-level communication between the two pathways, often involved in the occurrence of cellular senescence [202].

Hematopoietic stem cell/hematopoietic progenitor cell (HSC/HPC) aging is closely related to aging and a variety of senile diseases, ginsenoside Rg1 can inhibit telomere shortening, enhance telomerase activity, regulate the expression of aging-related genes and cell cycle-related proteins and treat Tert-butyl Hydroperoxide (TBHP)-induced HSC/HPC aging, and its mechanism of action is related to the regulation of SIRT6 and NF-κB (SIRT6 Enhanced expression of mRNA and protein [203].

In studies based on TBHP and psoralen, ultraviolet rays induced senescence in human diploid cells and human fibroblasts, respectively, ginsenoside Rg1 can delay cellular senescence by reducing p21 expression levels and enhancing telomerase activity [204, 205].

### *Panax ginseng* C. A. Meyer and its compounds delay cellular senescence by inhibiting oxidative stress

Ginseng and its compounds play an important role in the nervous system. In Alzheimer's disease-based studies on tree shrews, ginsenoside Rg1 may reduce oxidative stress response and improve cognitive impairment in tree shrews [185].

In studies based on cerebral infarction mice and D-Gal-induced neural stem cell aging, ginsenoside Rg1 can inhibit oxidative stress response through the Akt/mTOR signaling pathway, thereby delaying aging [206, 207]. In the study of mouse myocardial cells based on mesenchymal stem cells and hydrogen peroxide-stimulated rat bone marrow stem cells, HG/palmitate-stimulated rat cardiomyocytes, ginsenoside Rg1 can inhibit oxidative stress response by regulating the PI3K/AKT signaling pathway and enhancing NRF2 expression levels [208–210]. miR-144 directly regulates NRF2 production in neurodegenerative diseases [211]. In ischemia/reperfusion (I/R)-induced neuronal injury, ginsenoside Rg1 can inhibit oxidative stress by reducing miR-144 and enhancing NRF2 expression levels [212].

In addition, in studies of oxidative stress injury in cardiomyocytes based on liver injury mice and hypoxia/reoxygenation (H/R), ginsenoside Rg1 inhibited oxidative stress levels by enhancing NRF2 expression levels [213–215].

### *Panax ginseng* C. A. Meyer and its compounds delay cellular aging by improving autophagy

Ginseng can affect autophagy in the liver, lungs and heart, but there is no direct evidence that ginseng can delay cell aging by regulating autophagy. In carbon tetrachloride-induced mice with acute liver injury and paraquat-stimulated lung epithelial cells, ginsenoside Rg1 reduced the expression of inflammatory factors such as NF-κB and NLRP3 and enhanced autophagy [216, 217]. In starvation-induced cardiomyocyte stress, ginsenoside Rg1 increases autophagosome levels and enhances autophagy.

### *Panax ginseng* C. A. Meyer and its compounds delay cellular aging by improving mitochondrial disorders and DNA damage

Ginsenoside Rg1 can delay the aging of D-Gal-induced hematopoietic stem cell/progenitor cell mice via the p16^Ink4a^p53-p21 pathway. Ginseng and its compounds improve mitochondrial disorders and DNA damage in the heart, kidneys, and nervous system.

In lipopolysaccharide and glucose-stimulated cardiomyocyte stress response, ginsenoside Rg1 can improve mitochondrial disorders by increasing MMP, PINK1, AMPK levels [218, 219]. Siderozosis is a novel form of programmed cell death with iron-dependence, caused by lipid peroxidation and characterized by decreased GSH and glutathione peroxidase 4 (GPX4). In carbon tetrachloride-induced hepatic injury mice and lipopolysaccharide-induced human tubular epithelial cell siderozosis, ginsenoside Rg1 improves mitochondrial disorders by NRF2 and inhibition of the ferrotic pathway [220, 221].

In the nervous system, ginseno polysaccharides can regulate mitophagy by influencing the PINK1/Parkin pathway, maintain mitochondrial homeostasis, improve D-gal-induced nerve cell aging, and improve symptoms in Parkinson’s mice by improving mitochondrial disorders and DNA damage [61, 222].

Growth Arrest Specific 5 (GAS5), one of the long non-coding RNAs (lncRNAs), has been demonstrated to be involved in a number of physiological and pathological processes. GAS5 can lessen depressive-like behavior by preventing hippocampal neuronal damage [222].

### *Panax ginseng* C. A. Meyer and its compounds delay cellular aging by inhibiting the inflammatory response

Ginsenoside Rb1 can delay replicating endothelial cells by inhibiting NF-κB-p65-mediated inflammatory responses. Aging, reducing inflammatory factor IL-6, TNF-α levels, and NF-κB-p65 activity [223]. It can also play the role of anti-radiation injury-induced HSC/HPC aging by regulating the SIRT6/NF-κB signaling pathway [224].

In addition, ginsenoside Rb1 improves inflammation in the heart, kidneys, and nervous system.

In the nervous system, ginsenoside Rg1 can improve murine microglia and brain type I astrocytes damage through the NF-κB/TLR4 inflammatory signaling pathway and the p38/JNK-MAPK signaling pathway [225, 226]. In a spinal cord injury and cognitive impairment-based study in rats, ginsenoside Rb1 improves injury and cognitive impairment via NRF2 and PI3K/AKT inflammatory signaling pathways [227, 228]. Furthermore, in LPS-induced studies of mice with cardiomyocyte injury and chronic kidney injury, ginsenoside Rg1 reduced NLRP3 inflammatory factors and regulated NF-κB/TLR4 signaling pathway, thereby inhibiting inflammatory response [229]. In studies in aging mice, ginsenoside Rg1 reduced NOX4, NF-κB, NLRP3 inflammatory expression levels, thereby improving aging-induced liver fibrosis [230].

### *Panax ginseng* C. A. Meyer and its compounds delay cellular aging by regulating metabolism

Ginsenoside Rg1 plays an important role in the treatment of metabolic disorders and is associated with the regulation of AMPK (Li et al., 2018). Glucagon is involved in hepatic gluconeogenesis and plays an important role in maintaining the balance of glucose metabolism [231]. In studies of high-fat diet -fed mice/glucagon-challenged mice, ginsenoside Rg1 may block the glycolipid metabolism of dry mountain mice, by blocking the hepatic glucagon response [201]. In studies based on a high-fat diet and streptozotocin-induced T2DM rats, as well as methionine and choline deficiency -induced NASH mice, ginsenoside Rg1 reduced TC and TG levels in the above models [232–234].

### *Panax ginseng* C. A. Meyer and its compounds delay cellular aging by improving intestinal flora dysregulation

In studies on dextran sodium sulfate-induced colitis mice, ginsenoside Rg1 was observed to reduce the expression of Bacteroide, Ruminococcaceae by reducing M1/M2 values [235]. In a mouse study of dextran sodium sulfate-induced colitis, ginsenoside Rg1 increased MMP levels and reduced oxidative stress levels, and it was observed by bioprofiling that ginsenoside Rg1 modulates intestinal microbiota disorders in colitis mice [236]. Ginsenoside Rg1 can also regulate intestinal flora balance, boost the amount of good bacteria, and alleviate Alzheimer’s disease, according to studies in Alzheimer’s disease tree shrews. Symptoms of a tree shrew [237].

## Summary

**Figure 2:**
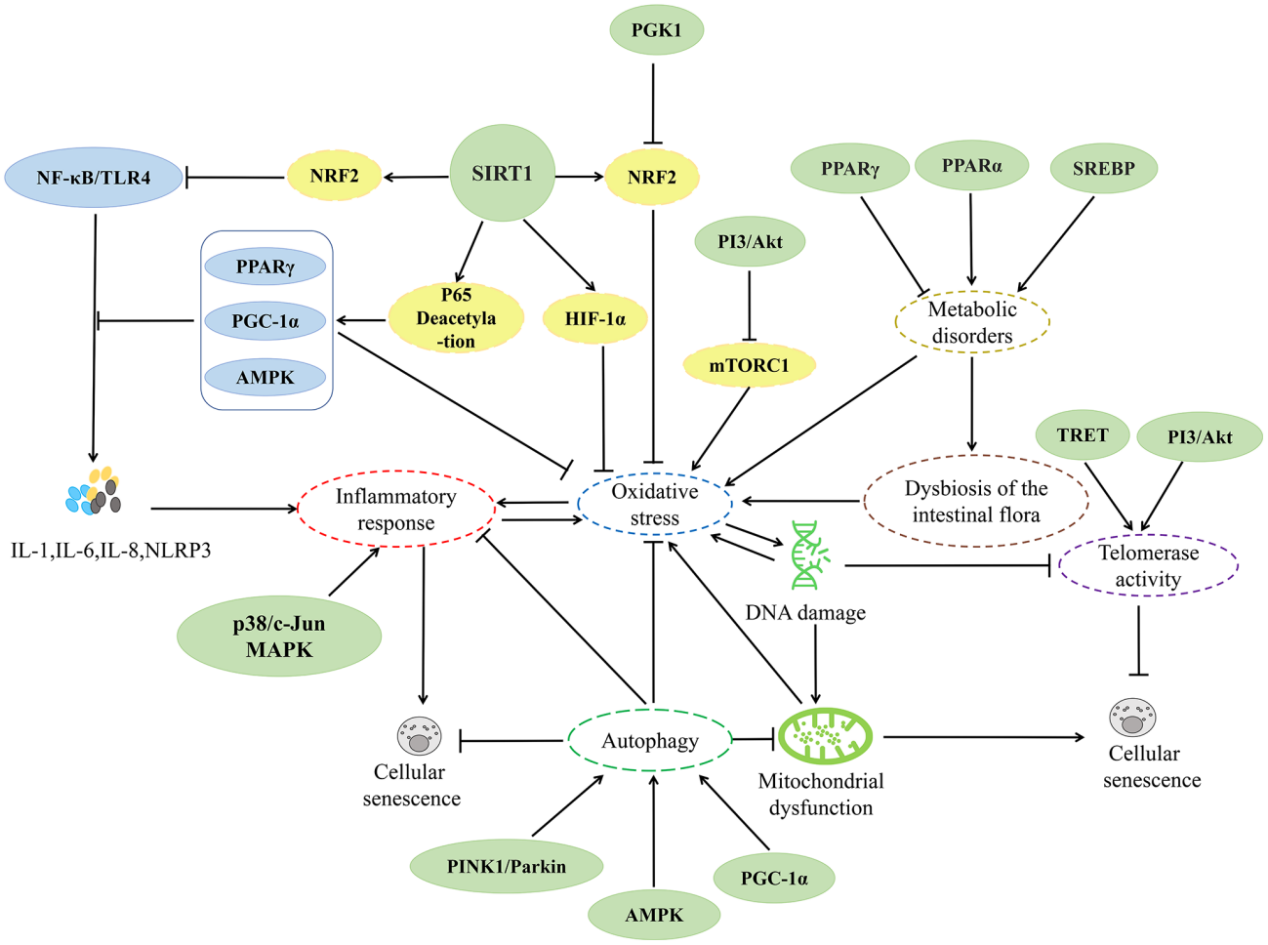
Relevant mechanisms affecting cellular senescence.

**Figure 3:**
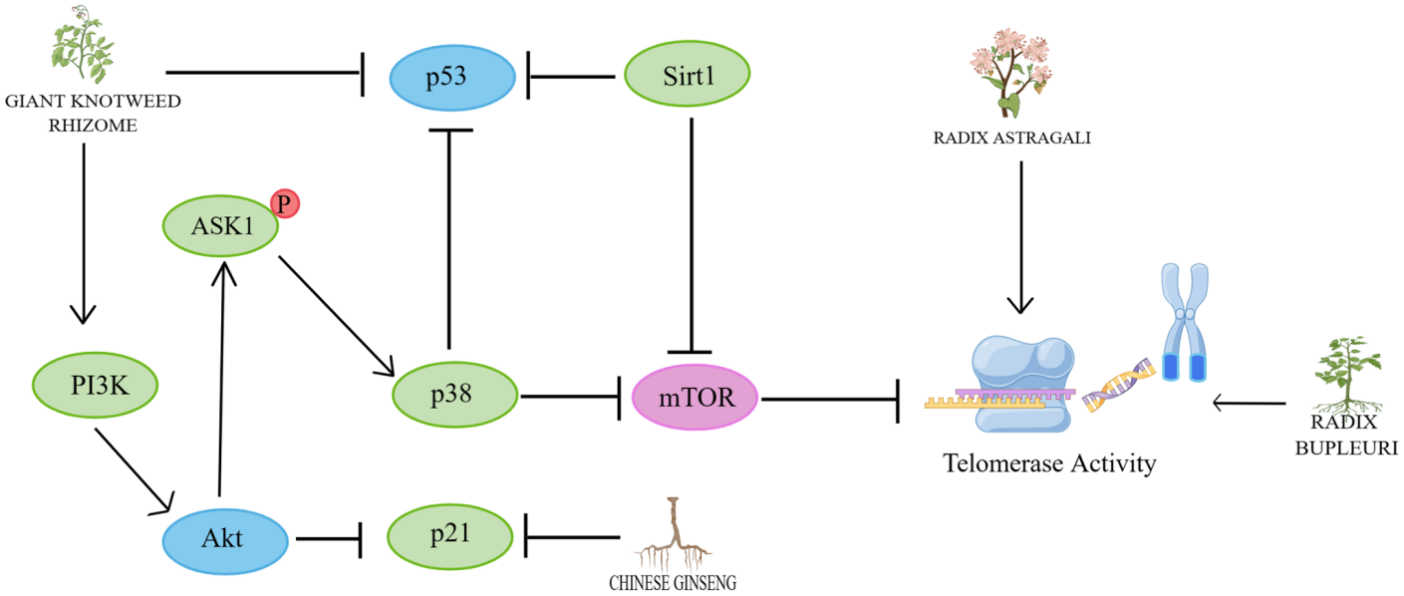
**Mechanisms affecting telomerase activity.** By Figdraw (http://www.figdraw.com).

**Figure 4:**
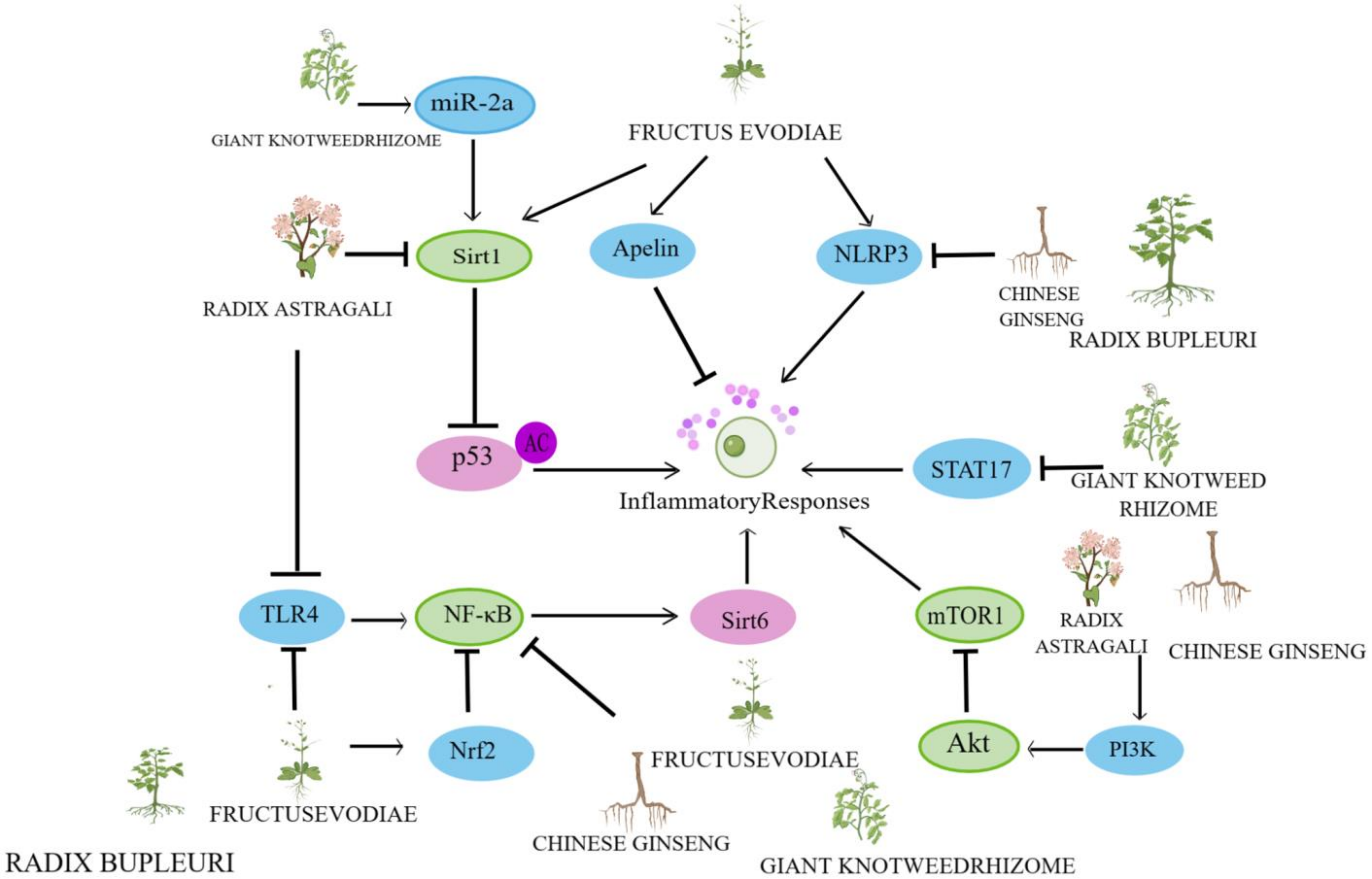
**Mechanisms affecting the oxidative stress response.** By Figdraw (http://www.figdraw.com).

**Figure 5:**
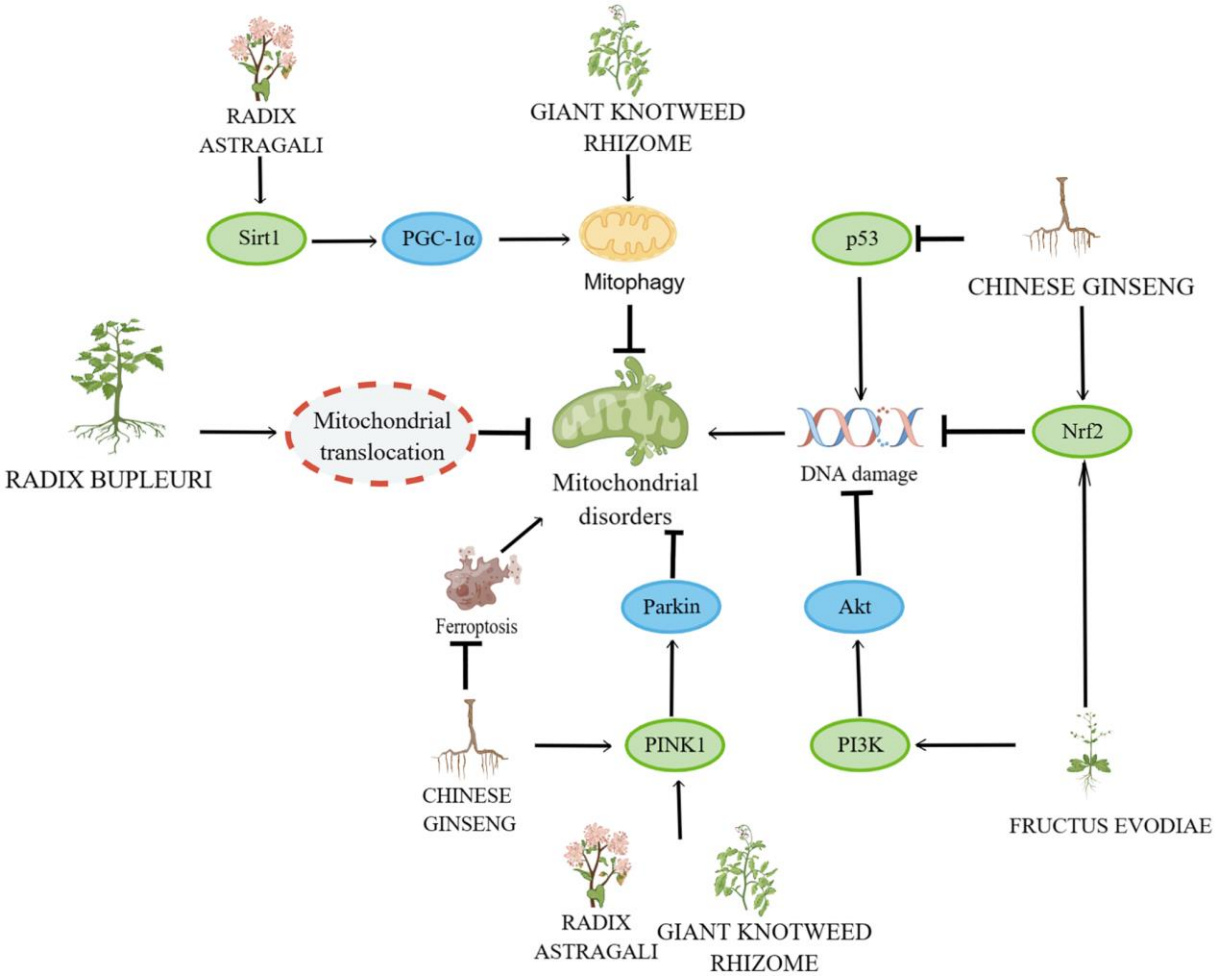
**Mechanisms affecting mitochondrial disorders and DNA damage.** By Figdraw (http://www.figdraw.com).

**Figure 6:**
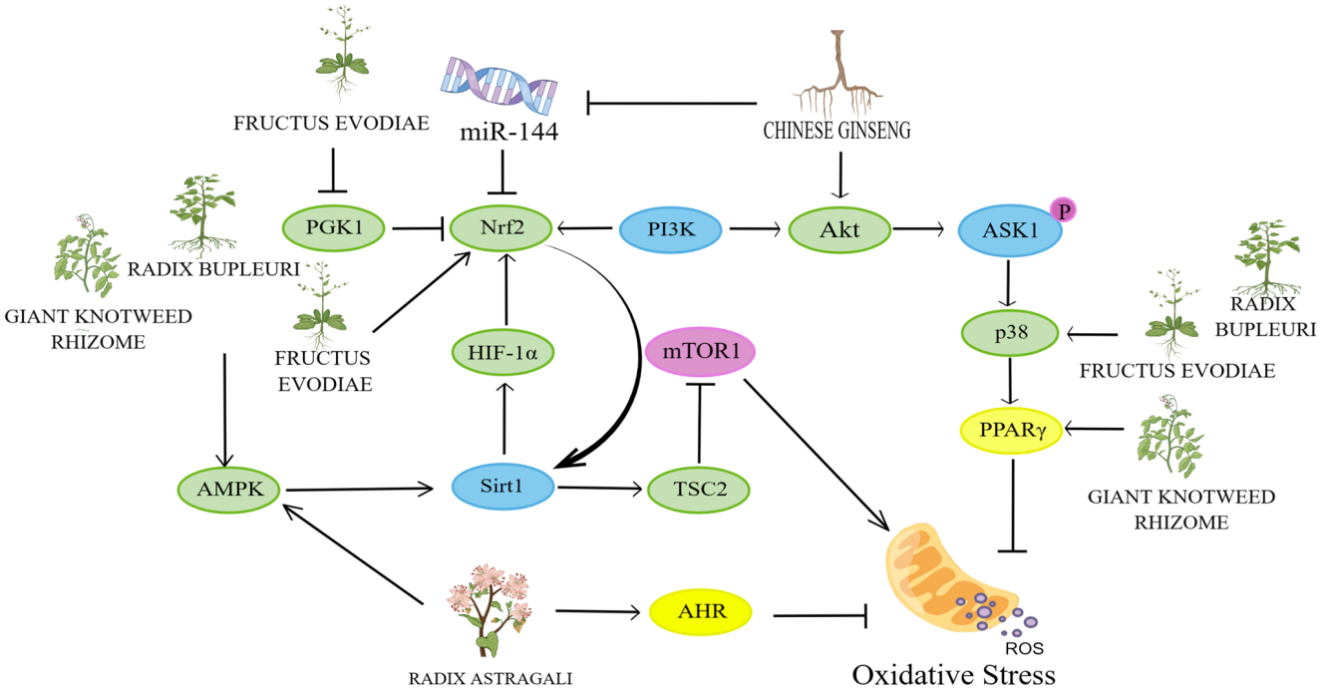
**Mechanisms affecting the inflammatory response.** By Figdraw (http://www.figdraw.com).

In recent years, research on herbs and their natural active ingredients for anti-aging has made significant progress. Various herbs impact multiple pathways involved in cellular senescence (Figure 2). The above content introduces important factors that affect cellular senescence, including mitochondrial dysfunction, DNA damage, telomere length shortening, imbalance of autophagy regulation, oxidative stress, and inflammatory response. This indicates that the specific mechanisms by which herbs regulate cellular senescence are closely associated with these factors (Figures 3–6).

However, in the current study, there are more reports on the direct impact of herbs on cellular senescence, while few reports focus on the specific mechanism by which herbs affect cellular senescence. However, there is still insufficient evidence from clinical trials and *in vivo* tests on the drugs and their active ingredients mentioned above. Therefore, additional *in vivo* trials and clinical evaluations of these active ingredients are required. Moreover, due to the limited number of studies investigating the treatment of various tissue and organ pathologies through metabolic regulation and improvement of intestinal dysbiosis, it is crucial to explore the association between herbal medicines and metabolism, intestinal dysbiosis, and other diseases.

Building upon the aforementioned, we propose that future investigations, both ours and those conducted by other researchers, should concentrate on the subsequent aspects: First, considering the impact of the aforementioned drugs on various cellular processes such as mitochondrial dysfunction, DNA damage, telomere length shortening, autophagy regulation imbalance, oxidative stress, and inflammatory response, our research aims to investigate the pathway from herbs/herbal extracts to cellular senescence mechanisms. This will help us understand the specific mechanism by which herbs prevent and treat cellular senescence, leading to the development of more effective anti-aging drugs and therapeutic interventions. Second, to study the active components in herbal extracts and discover and extract more extract components for more new research pathways. Third, it is important to shift the focus towards the chemical groups present in the active ingredients. By leveraging the shared characteristics of these chemical groups, we can develop superior and more effective compounds that can effectively delay cellular aging. Fourth, we will employ modern technologies, such as gene editing and genomics research, to investigate the regulatory effects of herbs on genes associated with cellular aging. This will enable us to uncover the molecular mechanisms by which herbs delay cellular aging and offer novel insights and methodologies for studying this process. Fifth, we will investigate the precise pathway involving herbs, gut flora, and aging, aiming to elucidate the specific mechanism by which herbs regulate cellular senescence through the gut flora. This research will contribute to the development of more potent drugs.

### Availability of data and materials

The data used and analyzed in this study are included within the article.

## Supplementary Materials

Supplementary Table 1
