# Supplementary material for: Traditional herbs: mechanisms to combat cellular senescence
Source: Aging (Albany NY). 2023 Dec 5;15(23):14473–505. doi: 10.18632/aging.205269 (PMC10756111; doi:10.18632/aging.205269)
Supplement: Supplementary Table 1 [file aging-15-205269-s001.docx]

**Supplementary Table 1. Herb-derived products with anti-aging effects on cells.**

| Drugs | Mechanism | Models | Chinese medicine or active ingredient | Targets/signalling pathways | References |
| --- | --- | --- | --- | --- | --- |
| *Astragalus membranaceus*（Fisch. ）Bunge. | Telomerase activity | Rat liver cells | Astragalus Membranaceus Total Extract | Telomerase activity,  TERT↑ | [14] |
|  |  | Nucleus pulposus cells | Cycloastragenol |  | [15] |
|  |  | Mouse embryonic fibroblasts |  |  | [16] |
|  | Oxidative stress | Rat liver cells | Astragalus Membranaceus Total Extract | TBA,8-OH-dG↓ | [14] |
|  |  | Mice |  | MDA,ROS↓  SOD↑  PI3K/Akt1 | [24] |
|  |  | Rat dermal fibroblasts | Astragaloside IV | ROS↓  SOD↑ | [15] |
|  |  | Human umbilical vein endothelial cells |  | SIRT1/AMPK | [29] |
|  |  | Human renal cortical proximal tubular epithelial barrier cells |  | ROS↓  AhR ↓ | [25] |
|  | Autophagy | Mouse macrophages | Astragalus Injection | Autophagosomes,IL-6↓  AMPK/mTOR | [35] |
|  |  | Rat lung tissue | AstragalosideIV | ROS,  Autophagosomes↓  SOD↑  PI3K / Akt /mTOR | [36] |
|  |  | Type 2 diabetic rats | Astragalus polysaccharide | IL-6↓  AMPK/mTOR | [22] |
|  |  | Cardiomyocyte-injured rats and mice with heart failure |  | Autophagosomes↓  AMPK/mTOR | [37] |
|  | Mitochondrial disorders and DNA damage | Human renal cortical proximal tubule epithelial cells | Formononetin | ROS↓  Sirt1/PGC-1α ↑ | [43] |
|  |  | Glial cells | Astragaloside | ROS↓  PINK1/Parkin ↓ | [45] |
|  |  | Human monocytes | Ononin | Mitochondrial autophagy↑ | [46] |
|  |  | Rat aortic endothelial cells | Astragalus polysaccharide | Na^+^/Ca^2+^↑  NLRP3,IL-1β,p16,p21,  p53↓ | [47] |
|  | Inflammatory response | Mice with idiopathic pulmonary fibrosis | AstragalosideIV | IL-6↓  TLR4/NF-κB ↓ | [57] |
|  |  | Human renal cortical proximal tubule epithelial cells |  | IL-6↓  SITR1/p53↑ | [52] |
|  |  | Mouse asthma model |  | IL-4,IL-5,IL-17,  mTORC1↓  AKT/mTOR | [23] |
|  |  | Rat synovial fibroblasts | Astragalus polysaccharide | IL-6↓  PI3K/AKT/mTOR | [53] |
|  |  | Rat aortic endothelial cells |  | Na^+^/Ca^2+^↑  NLRP3,IL-1β,p16,p21,p53↓ | [47] |
|  | Metabolism | Type 2 diabetic mice | AstragalosideIV | ROS,MDA↓,  SOD↑  PI3K/AKT,AMPK/sirt1 | [62] |
|  |  |  | Astragalus polysaccharide | ROS,IL-6,Shigella bacteria↓  Heterotrophic bacteria,Lactobacillus↑ | [61] |
|  |  |  |  | T1R2,Gα,TRPM5↑ | [64] |
|  |  | Rats with non-alcoholic fatty liver disease |  | TLR4,NF-κB,NLRP3,  Thick-walled Bacteria / Bacteroidetes↓  Bacillus deformans↑ | [72] |
|  | Intestinal flora | Mice with idiopathic pulmonary fibrosis |  | IL-6↓  TLR4/NF-κB | [57] |
|  |  | Type 2 diabetic mice |  | ROS,IL-6,  Shigella bacteria↓  Heterotrophic bacteria,  Lactobacillus↑ | [61] |
|  |  |  |  | STR↑ | [64] |
|  |  | Cholesterol gallstones in mice |  | Phyllostomycetes↑ | [73] |
| *Reynoutria japonica* Houtt.(R. japonica) | Telomerase activity | Human myeloid cells | Resveratrol | p53↓  TERT↑ | [81] |
|  |  | Endothelial progenitor cells |  | Telomerase ↑  PI3K-Akt | [82] |
|  | Oxidative stress | Bone marrow stromal stem cells |  | ROS↓  PPAR-γ↑ | [88] |
|  |  |  |  | ROS,P53,P16,  P21↓  SOD↑,  MAPK | [84] |
|  |  | Vascular smooth muscle cells |  | SOD↑,  AMPK/sirt1 | [83] |
|  |  | Glomerular foot cells | Polydatin | ROS↓,  HIF-1α/NOX4 | [97] |
|  |  | Human retinal epithelial cells | Polysaccharide | ROS↓  SOD,NRF2↑ | [98] |
|  |  | Cardiac senescence in mice |  | ROS,p53↓  SOD↑ | [99] |
|  |  | Renal aging mice |  | AKt,MDA↓  SOD↑ | [100] |
|  |  | Liver-damaged mice | Aqueous extract of Tigernut | NRF2,SOD↑  MDA↓ | [101] |
|  | Autophagy | Human umbilical vein endothelial cells | Resveratrol | Notch1,mTOR↓ | [110] |
|  |  | Myogenic cells |  | P16,P21↓  Autophagosomes↑ | [102] |
|  |  | Atherosclerotic mice | Polydatin | NLRP3,mTOR,  IL-1β,  IL-18↓  Autophagosomes↑ | [107] |
|  | Mitochondrial disorders and DNA damage | Human umbilical vein endothelial cells/Human myeloid cells | Resveratrol | ROS↓  Autophagosomes,SOD ↑ | [106] |
|  |  | Human embryonic lung fibroblasts/human peritoneal mesothelial cells |  | ROS,p53↓  SOD↑ | [104] |
|  |  | Human nasal epithelial cells/allergic rhinitis in mice | Polydatin | NLRP3,ROS↓  PINK1-Parkin | [104] |
|  | Inflammatory response | Human airway epithelial cells | Resveratrol | SIRT1↑  IL-1β,IL-6,  IL-8,NF-κB,  p65↓ | [110] |
|  |  | Osteoarthritic rats/rat chondrocytes | Polydatin | NF-κB,IL-6,  MMP↓ | [112] |
|  |  | Mice with colitis |  | STAT17,Th3↓ | [114] |
|  | Metabolism | Human umbilical vein endothelial cells | Polydatin | miR-26a-5p/BID | [116] |
|  |  | Insulinoma cells/diabetic mice |  | TG,TC↓ | [118] |
|  |  | Fat cells | Polysaccharide | IL-1β,IL-6↓  NRF2↑ | [119] |
|  |  | Obese mice |  | IL-1,IL-1β,  SREBP-1,  PPARα↓  AMPK↑ | [122] |
|  |  | Hyperlipidemic mice |  | TG,TC,PPARα,  SREBP1c↓ | [123] |
|  |  | Type 2 diabetic mice | Saponin | Enterococcus,  Enterobacteria-  ceae,  TG,TC,LDL↓  AKT,Bifidobacte  -rium bifidum,Lactobacillus↑ | [124] |
|  |  |  | Aqueous extract of Tigernut | TG,TC,LDL↓  PI3K/AKT | [125] |
|  | Intestinal flora | Metabolic syndrome rats | Resveratrol | Enterobacte  -riaceae,  Streptococcus↓  Bifidobacterium-bifidum,Lactobac-  illus↑ | [128] |
|  |  | Obese mice |  | ROS  Heterobacterium spp. ↑ | [127] |
|  |  | Type 2 diabetic mice | Saponin | Enterococcus,  Enterobacteria  -ceae,  TG,TC,ox-LDL↓  AKT,  Bifidobacterium- bifidum,Lactob  -acillus↑ | [124] |
|  |  | Mice | Morinda Citrifolia Ethanol Extract | Lactobacillus spp.  ,Enterobacteriacea-e↑ | [126] |
| *Bupleurum scorzonerifolium*. | Telomerase activity | Human aortic endothelial cells | Quercetin | ox-LDL↓Telomerase↑ | [133] |
|  |  | Vascular smooth muscle cells |  | ROS,MDA↓  AMPK,SOD↑ | [134] |
|  | Oxidative stress | Lung epithelial cells | Chai Hu polysaccharide | ROS,MDA↓  SOD↑ | [135] |
|  |  | Nerve cellsNerve cells | SaikosaponinD | ROS,MDA↓  SOD↑  PI3K/NRF2 | [136] |
|  |  | Chondrocytes |  | IL-6,ROS↓  NRF2↑ | [137] |
|  |  | Cardiomyocyte inflammation |  | ROS,MDA↓  p38-MAPK | [138] |
|  | Autophagy | Liver fibrosis mice | Quercetin | p62↑ | [140] |
|  |  | Myeloid nucleus |  | ROS↓  Autophagoso  -mes ↑  p38MAPK / mTOR | [139] |
|  | Mitochondrial disorders and DNA damage | Rat neuronal cells neuronal cells | SaikosaponinD | NF-κB↓ | [141] |
|  |  | Human dermal fibroblasts | Quercetin | p16^INK4A^, p53↓ | [27] |
|  | Inflammatory response | Microglia/neuritis mice | Ethanolic extract of Radix Bupleurum | IL-6,IL-1β,  ROS,NF-κB↓ | [142] |
|  |  | Nasopharyngeal carcinoma senescent cells | Quercetin | IL-6,IL-1β,  NF-κB↓  NRF2↑ | [143] |
|  |  | Pulmonary inflammation in mice | SaikosaponinA | IL-1β,NF-κB↓  MDA,NRF2↑ | [144] |
|  |  | Mice with endometritis |  | IL-6,IL-1β,  NF-κB↓  NRF2↑ | [145] |
|  |  | Mice with acute lung injury |  | IL-6,IL-1β↓  NF-κB/TLR4 | [146] |
|  |  | Macrophages | SaikosaponinB | IL-6,IL-1β,  NF-κB↓ | [147] |
|  |  | Liver damaged mice |  | IL-6,IL-1β,  NF-κB↓ Sirt-6,Na^+^-K^+^-ATPPase,  Ca^2+^-Mg^2+^-ATPPase↑ | [148] |
|  |  | Depressed mice | SaikosaponinC | IL-6,IL-1β↓  Microglia↑ | [152] |
|  |  | Diabetic rats |  | IL-6,IL-1β↓  AQP1 / RhoA / ROCK | [149] |
|  |  | Microglia/mouse neuroinflammation | SaikosaponinD | IL-6,IL-1β↓  NF-κB/TLR4 | [153] |
|  |  | Liver fibrosis mice |  | IL-β,NLRP3↓ | [154] |
|  |  |  |  | ROS,MMP,  NLRP3↓ | [155] |
|  |  |  |  | MDA,NLRP3,  IL-1β↓  SOD↑ | [156] |
|  | Metabolism | Hyperlipidemic rats | Saikosaponin | TC,TG↓ | [154] |
|  |  | Hyperlipidemic pancreatitis in rats | SaikosaponinA | PPARγ↑  IL-6,IL-1β,  NF-κB,TC↓ | [157] |
|  |  | Metabolic fatty liver mice | SaikosaponinD | PPARα,TG,SREBP1c↓ | [158] |
|  |  | Liver-damaged mice | Saikosaponinb2 | IL-6,IL-1β,NF-κB↓ Sirt6,Na^+^-K^+^-ATPPase,  Ca^2+^-Mg^2+^-ATPase ↑ | [148] |
|  | Intestinal flora | Rats with severe acute pancreatitis | SaikosaponinA | SOD↓  Prevotella,  lactic acid bacteria↑  Keap1/NRF2/ARE | [159] |
|  |  | Colitis mice | SaikosaponinD | IL-6,IL-1β,  NF-κB,  Ruminiclostridium↓  Roseburia,Helicobater,  Lactobacillus↑ | [160] |
| *Evodia rutaecarpa* (Juss.) Benth. | Oxidative stress | Brain damaged mice | Corydaline | ROS↓SOD↑  PGK1 / NRF2 | [166] |
|  |  | Endothelial cells | Rutinine | p21,ROS↓  Cα,SIRT1↑ | [167] |
|  |  | Cardiomyocytes |  | IL-6,IL-1β,ROS,MDA↓  SOD↑  MAPK | [173] |
|  |  | Cardiotoxic mice |  | MDA↓  SOD,NRF2↑ | [174] |
|  |  | Inflammatory bowel disease in mice |  | ROS↓  NRF2↑ | [164] |
|  |  | Cerebral ischemia/reperfusion in rats |  | IL-6, IL-1β,SOD↓  NRF2↑ | [169] |
|  |  | Gastric mucosal injury in mice |  | IL-6,IL-1β,MDA↓  SOD,PI3K,  NRF2↑ | [170] |
|  |  | Renal ischemia-reperfusion in mice |  | p38,c-Jun,IL-6, IL-1β  ,NF-κB,MDA,  ROS↓  SOD↑ | [172] |
|  | Autophagy | Astrocytes | Corydaline | Autophagosomes↑  JNK / p38 MAPK | [175] |
|  |  | Colitis mice |  | IL-1β,NFκB,NLRP3↓  Autophagosomes↑ | [176] |
|  | Mitochondrial disorders and DNA damage | Mouse liver cells | Rutinine | JNK / p38 MAPK ,PI3K/Akt | [177] |
|  |  | Endothelial inflammation |  | NF-κB↓  NRF2↑ | [178] |
|  |  | Liver cells |  | ROS↓  NRF2↑ | [179] |
|  |  | Liver-damaged mice |  | IL-1β,IL-6,MDA↓  NRF2↑ | [181] |
|  | Inflammatory response | Colitis mice | Corydaline | IL-1β,NFκB,NLRP3↓  Autophagosomes↑ | [182] |
|  |  |  |  | IL-1β,IL-6,NF-κB,NLRP3,Enterobacteriaceae↓  Lactobacillus↑ | [183] |
|  |  | Mouse mammary epithelial cells |  | IL-1β,NF-κB,p38,JNK,AKT↓  p38/JNK-MAPK | [184] |
|  |  | Asthmatic mice |  | NF-κB/TLR-4 | [183] |
|  |  | Human umbilical vein endothelial cells |  |  | [184] |
|  |  | Rat kidney cells |  | ROS,NF-κB↓ | [187] |
|  |  | Mouse microglia |  | IL-6,NF-κB ↓  AKT/NRF2 | [188] |
|  |  | Pulmonary inflammation/fibrosis in mice |  | IL-6↓  ERK1/2↑ | [190] |
|  |  | Human myeloid cells |  | IL-6↓  SIRT1↑PI3K / Akt | [189]  [191] |
|  |  | Acute pancreatitis in rats | Rutinine | IL-6↓  MAPK/NF-κB | [191] |
|  |  | Macrophages |  | IL-1β,NF-κB,MDA↓  p38/JNK-MAPK | [186] |
|  |  | Hepatotoxic mice |  | IL-6,IL-1β,  NF-κB,MDA↓  NRF2↑ | [181] |
|  | Metabolism | Type 2 diabetic rats | Corydaline | TC,TG,MDA,  IL-6,IL-1β↓ | [194] |
|  |  | Hyperlipidemic mice |  | TC,TG↓  PPARγ↑ | [196] |
|  |  | Obese/diabetic mice |  | AMPK/mTOR | [198] |
|  |  | Hyperlipidaemic/hyperglycaemic rats |  | TC,TG,NF-κB,IL-6↓  PI3K / Akt,AMPK↑ | [195] |
|  |  | Myocardial infarction rats |  | PPARα↓  ATP↑ | [197] |
|  |  | Obese mice |  | TG,PPARα↓ | [178] |
|  | Intestinal flora | Liver fibrosis mice | Corydaline | IL-6,IL-1β,Enterococcu,  Rachnochia↓  Lactobacillus  ,Ackermaniaspp.,  Mycobacterium avium↑ | [199] |
|  |  | Colitis mice |  | IL-1β ,  Thick-walledbacteria/bacteroids,Alloprevotella, Lachnoclostridium, Oscillibacter, Lachnospiraceae,Ruminococcaceae, Bacteroidales. ↓  Bacteroides, Parasutterella, Turicibacter↑ | [198] |
|  |  |  |  | NLRP3,IL-1β,IL-6,  NF-κB,  Enterobacteriaceae↓  Lactobacillus↑ | [182] |
| *Panax ginseng*C.A.Mey. | Telomerase activity | Human diploid cells | Ginsenoside Rg1 | P21↓ | [204] |
|  |  | Human fibroblasts |  | P21↓  Telomerase activity↑ | [205] |
|  |  | Aging rats |  | IL-6,IL-1β↓  SOD,Telomerase↑ | [203] |
|  |  | Mesenchymal stem cells |  | NRF2↑  PI3K/AKT | [209] |
|  |  | Neuronal damage |  | ROS,miR-144↓  NRF2↑ | [212] |
|  |  | Haematopoietic stem cells / haematopoietic progenitor cells | Ginsenoside Rg1 | SIRT6,NF-κB↓  Telomerase activity↑ | [203] |
|  | Oxidative stress | Neural stem cell aging | Ginsenoside Rg1 | ROS,MDA,p53,p16,p21↓  SOD↑  Akt/mTOR | [206] |
|  |  | Mice with cerebral infarction |  | HIF-1α↓  PI3K/Akt/mTOR | [207] |
|  |  | Rat Bone Marrow Stem Cells |  | PI3K / AKT | [208] |
|  |  | Mesenchymal stem cells |  | NRF2↑  PI3K/AKT | [209] |
|  |  | Rat cardiomyocytes |  | ROS,MDA↓  SOD,NRF2↑  PI3K / AKT | [210] |
|  |  | Neuronal damage |  | ROS,miR-144↓  NRF2↑ | [212] |
|  |  | Cardiomyocytes |  | ROS,SOD,c-Jun ↓  NRF2↑ | [214] |
|  |  | Hepatotoxic mice |  | SOD↓  NRF2↑ | [215] |
|  |  | Liver-damaged mice |  | ROS,MDA↓  NRF2↑ | [213] |
|  |  | Alzheimer's disease tree shrew |  | MDA,IL-1β↓  SOD↑ | [216] |
|  | Autophagy | Liver-damaged mice | Ginsenoside Rg1 | IL-6,IL-1β, NF-κB,NLRP3↓ | [217] |
|  |  | Lung epithelial cells |  | IL-6,IL-1β↓ | [216] |
|  |  | Cardiomyocytes |  | Autophagosomes↑ | [218] |
|  | Mitochondrial disorders and DNA damage | Chronic Restraint Stress Depression in Rats | Ginsenoside Rg1 | IL-6,IL-1β,ROS,GAS5↓  MMP,NRF2↑ | [218] |
|  |  | Cardiomyocyte model/septic mice |  | ROS↓  MMP↑ | [219] |
|  |  | Cardiomyocytes |  | PINK1,AMPK↑ | [217] |
|  |  | Chronic Parkinson's disease in mice |  | ROS↓  Mitochondrial disorders and DNA damage | [225] |
|  |  | Aging mice |  | ROS,MDA↓  SOD↑p16^Ink4a^p53-p21 | [226] |
|  |  | Liver-damaged mice |  | MDA,IL-6,IL-1β↓  SOD,NRF2↑ | [221] |
|  |  | Human renal tubular epithelial cells |  | IL-1β,MDA↓  SOD↑ | [220] |
|  |  | Nerve cellsNerve cells | Ginseng polysaccharide | PINK1/Parkin | [217] |
|  | Inflammatory response | Cardiomyocytes | Ginsenoside Rg1 | IL-1β,MMP,NLRP3↓  NF-κB/TLR4 | [229] |
|  |  | Haematopoietic stem cells / haematopoietic progenitor cells |  | SIRT6,NF-κB↓ | [230] |
|  |  | Endothelial cells |  | NF-κB,IL-6,p65↓ | [223] |
|  |  | Microglia |  | IL-1β,IL-6↓  NF-κB/TLR4 | [226] |
|  |  | Spinal cord injury |  | IL-1β,IL-6,MDA↓  SOD,NRF2↑ | [227] |
|  |  | Cognitively impaired rats |  | IL-1β,IL-6,MDA,  p21,p53↓  PI3K / AKT | [228] |
|  |  | Mice with chronic kidney injury |  | IL-1β,ROS,NLRP3↓ | [230] |
|  |  | Aging mice |  | ROS,NOX4,  NF-κB,NLRP3↓ | [232] |
|  |  | Rat brain type I astrocytes |  | p38/JNK-MAPK | [232] |
|  | Metabolism | High fat diet -fed mice/glucagon attacked mice | Ginsenoside Rg1 | TG,TC,Akt↓ | [219] |
|  |  | Obese mice |  | AMPK↑ | [218] |
|  |  | Diabetic rats |  | TG,TC,IL-1β,IL-6,NF-κB↓ | [233] |
|  |  | Type 2 diabetic rats |  | TC, TG↓ | [234] |
|  |  | NASH Mice |  | TC,TG,ROS,  IL-6↓ | [232] |
|  | Intestinal flora | Colitis mice | Ginsenoside Rg1 | IL-6,M1/M2,  Bacteroide,  Ruminococcaceae↓ | [235] |
|  |  | Colitis mice |  | ROS↓  MMP↑ | [236] |
|  |  | Alzheimer's disease tree shrew |  | Firmicutes/Bacteroides,  Lactobacillus salivarius↑  Bacteroidetes↓ | [237] |
